# Photodynamic and nitric oxide therapy-based synergistic antimicrobial nanoplatform: an advanced root canal irrigation system for endodontic bacterial infections

**DOI:** 10.1186/s12951-024-02483-8

**Published:** 2024-04-30

**Authors:** Youyun Zeng, Xiangyu Hu, Zhibin Cai, Dongchao Qiu, Ying Ran, Yiqin Ding, Jiayi Shi, Xiaojun Cai, Yihuai Pan

**Affiliations:** School and Hospital of Stomatology, Wenzhou Medical University, Wenzhou, 325027 China

**Keywords:** Antimicrobial photodynamic therapy, Nitric oxide gas therapy, Root canal irrigation, Multifunctional nanoparticles, Biofilm, Antimicrobials, Osteogenesis

## Abstract

**Background:**

The main issues faced during the treatment of apical periodontitis are the management of bacterial infection and the facilitation of the repair of alveolar bone defects to shorten disease duration. Conventional root canal irrigants are limited in their efficacy and are associated with several side effects. This study introduces a synergistic therapy based on nitric oxide (NO) and antimicrobial photodynamic therapy (aPDT) for the treatment of apical periodontitis.

**Results:**

This research developed a multifunctional nanoparticle, CGP, utilizing guanidinylated poly (ethylene glycol)-poly (ε-Caprolactone) polymer as a carrier, internally loaded with the photosensitizer chlorin e6. During root canal irrigation, the guanidino groups on the surface of CGP enabled effective biofilm penetration. These groups undergo oxidation by hydrogen peroxide in the aPDT process, triggering the release of NO without hindering the production of singlet oxygen. The generated NO significantly enhanced the antimicrobial capability and biofilm eradication efficacy of aPDT. Furthermore, CGP not only outperforms conventional aPDT in eradicating biofilms but also effectively promotes the repair of alveolar bone defects post-eradication. Importantly, our findings reveal that CGP exhibits significantly higher biosafety compared to sodium hypochlorite, alongside superior therapeutic efficacy in a rat model of apical periodontitis.

**Conclusions:**

This study demonstrates that CGP, an effective root irrigation system based on aPDT and NO, has a promising application in root canal therapy.

**Supplementary Information:**

The online version contains supplementary material available at 10.1186/s12951-024-02483-8.

## Introduction

Apical periodontitis (AP) is an inflammatory condition primarily caused by bacterial infections, leading to the destruction of alveolar bone [1]. If not properly treated, AP can escalate into serious complications such as cellulitis, jaw osteomyelitis, and sepsis [2]. Notably, the worldwide prevalence of AP is reported to be approximately 52% [3]. Conventional root canal therapy, aimed at addressing AP, involves the removal of infected pulp tissue and microorganisms, followed by disinfection and filling of the root canal with an inert material. This process typically employs mechanical preparation and chemical irrigation for infection control. However, the complex anatomy of the root canal system, along with the persistent presence of biofilms, significantly hinders the effectiveness of these treatments. The failure rate of root canal treatment is 10–62% [4]. Consequently, the development of effective AP treatments is crucial.

Root canal treatment failures are primarily due to microbial retention, which resists disinfection efforts and form biofilms in the root canals [5]. Meanwhile, these biofilms protect bacteria by hindering the penetration of therapeutic agents and fostering an environment conducive to increased drug resistance [6]. Sodium hypochlorite (NaClO), the preferred root canal irrigant in clinics, possesses potent antimicrobial properties and tissue dissolution ability [7]. However, inadvertent leakage of NaClO beyond the root canal may lead to serious complications, including acute inflammatory injury, mucosal necrosis, osteonecrosis, and potentially life-threatening airway obstruction [8, 9]. In AP, pathogens-induced irritation of the apical portion often results in the resorption and destruction of adjacent alveolar bone [10]. Effectively controlling the infection and facilitating the repair of alveolar bone defects to shorten disease duration is challenging. Therefore, the development of safe and controllable root canal irrigation systems that can efficiently penetrate and eradicate biofilms, as well as promote alveolar bone repair, is of paramount importance.

Antimicrobial photodynamic therapy (aPDT) is an efficient, non-invasive antimicrobial strategy, which offers precise temporal and spatial control, broad-spectrum antimicrobial and biofilm eradication properties [11]. It operates by activating photosensitizers with lasers at specific wavelengths, leading to the generation of reactive oxygen species (ROS), such as hydroxyl radicals, superoxide anion, hydrogen peroxide (H_2_O_2_), and singlet oxygen (^1^O_2_). These ROS are lethal to bacteria, causing damage to cell membranes, proteins, lipids, and nucleic acids [12, 13]. Demonstrating effectiveness in inhibiting biofilms of burn wounds, chronically infected diabetic ulcers, and oral biofilms, aPDT holds promise for treating AP [14–16]. However, its efficacy is challenged in the hypoxic microenvironments of biofilms due to its oxygen-dependent nature [17, 18]. Furthermore, most conventional organic photosensitizers are hydrophobic and prone to aggregation in physiological conditions, reducing the ROS yield [19, 20]. Moreover, the use of high concentrations of photosensitizers in aPDT can result in the production of excessive ROS. This elevated ROS concentration may lead to a persistent inflammatory response, which, in turn, can adversely affect the process of osteogenesis [21, 22]. To overcome these challenges, research has focused on combining aPDT with alternative therapeutic approaches [23, 24]. This study explores the combination of aPDT with gas therapy, aiming to enhance biofilm eradication efficacy and mitigate the detrimental effects of elevated ROS levels.

Gas therapy, particularly with nitric oxide (NO), an endogenous gas molecule, has recently garnered attention as a green therapeutic strategy, acclaimed for its mild toxicity and non-resistance properties [25, 26]. NO plays a multifaceted role, contributing to anti-infection, inflammation regulation, and osteogenesis [27–30]. As a potent antimicrobial, NO interacts with the free radical superoxide to produce reactive byproducts, which lead to antimicrobial effects via lipid peroxidation, DNA damage, and protein dysfunction [29, 31]. Additionally, NO is known to stimulate osteogenesis by enhancing osteoblast activity [32]. Studies have shown that osteoblastic cell line MC3T3-E1 or pulp cells, when treated with NO donors, exhibit increased expression of osteogenic markers [33, 34]. Therefore, NO's dual capability to efficiently combat microbial presence and promote bone defect repair presents a promising avenue in the management of AP.

In our previous study [13], we demonstrated that dendritic peptide rich in L-arginine are capable of acting as macromolecular NO donors, releasing NO in response to oxidation by H_2_O_2_. Furthermore, the presence of the guanidino group in L-arginine significantly enhances the biofilm penetration ability of these dendritic peptides. Building on these insights, we propose that nanoparticles modified with guanidino groups could similarly function as NO donors and exhibit exceptional biofilm permeability. This concept underpins our approach in developing an effective root canal irrigation system, leveraging these properties to combat infections in endodontic treatments.

**Scheme 1:**
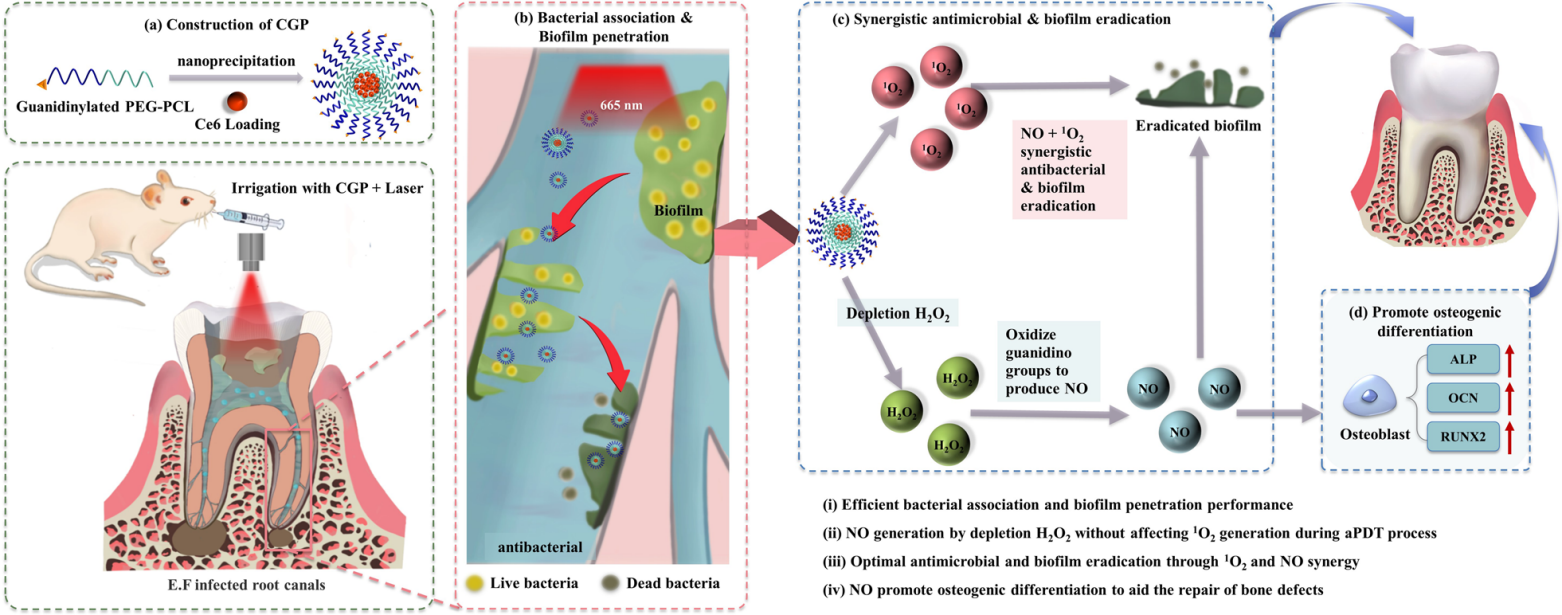
CGP preparation schematic and synergistic PDT/NO antibacterial effect. Schematic representation of CGP preparation, accompanied by an elucidation of the mechanisms underlying the highly efficient synergistic antibacterial action of PDT/NO and its role in promoting apical periodontitis healing

In the present study, we developed and synthesized guanidino-functionalized poly (ethylene glycol)-poly(ε-caprolactone) (PEG-PCL) block copolymers and used them to form chlorin e6 (Ce6)-loaded nanomicelles, named CGP (Scheme 1). These CGP nanomicelles are specifically designed to penetrate biofilms effectively during the root canal irrigation process. Leveraging the H_2_O_2_ produced by Ce6 in the aPDT process, CGP is engineered to oxidize and release NO. This NO release is expected to synergize with the ROS generated during aPDT, resulting in potent antibacterial activity and efficient biofilm eradication. Additionally, following the successful antibacterial action and biofilm removal, CGP nanomicelles are anticipated to aid in the repair of periapical bone defects, thereby significantly enhancing the overall treatment efficacy.

The combination of aPDT and NO offers a promising pathway for developing a new multifunctional root canal irrigation system. This study is designed to assess the antibacterial, biofilm eradication, and osteogenic differentiation capabilities of CGP through colony counting, live/dead bacterial viability assays, simulated root canal irrigation, and osteogenic differentiation experiments, along with therapeutic testing in a rat AP model. Compared to NaClO, CGP is anticipated to possess not only antimicrobial properties but also superior temporal and spatial control abilities, as well as a unique capacity to facilitate the repair of apical bone defects. We anticipate that this study has developed an irrigation system capable of safely and effectively treating AP.

## Materials and methods

### Materials

Amino-terminated poly (ethylene glycol)-poly(ε-caprolactone) (NH_2_-PEG-PCL) was obtained from Ponsure (Shanghai, China). Ce6 was obtained from Frontier Scientific (USA). 1-Amidinopyrazole hydrochloride, N, N-diisopropylethylamine, dichloromethane, glutaraldehyde, ethanol, methanol, cetylpyridinium chloride, crystal violet, acetic acid, and NaClO were purchased from Aladdin (Shanghai, China). Horseradish peroxidase, ethylenediaminetetraacetic acid disodium salt, phosphate-buffered saline (PBS), normal saline (NS), hematoxylin–eosin (HE) stain kit, and the Tartrate-resistant acid phosphatase (TRAP) stain kit were obtained from Solarbio (China). Dimethyl sulfoxide, β-glycerophosphate, and ascorbic acid were obtained from Sigma‒Aldrich (USA). The Griess reagent, H_2_O_2_ assay kit, singlet oxygen sensor green (SOSG), 2′7′-Dichlorodihydrofluorescein diacetate (DCFH-DA), 3-Amino,4-aminomethyl-2',7'-difluorescein, diacetate (DAF-FM DA), BCA protein assay kit, enhanced adenosine 5′-triphosphate (ATP) assay kit, Amplex red, Calcein/PI cell viability assay kit, BCIP/NBT alkaline phosphatase (ALP) color development kit, Alizarin red S staining kit and cell counting kit-8 were acquired from Beyotime (China). The osteoblastic cell line MC3T3-E1 and *Enterococcus faecalis* (*E. faecalis*, ID: ATCC29212) were purchased from the American Type Culture Collection (ATCC). The human periodontal ligament stem cells (hPDLSCs) were purchased from Procell Life Science and Technology (China). Minimum essential medium alpha modification (MEM α), Dulbecco's Modified Eagle Medium (DMEM), and fetal bovine serum were obtained from Gibco (USA). Enhanced RIPA lysis buffer, mouse osteocalcin (OCN) enzyme-linked immunosorbent assay (ELISA) kit, and mouse Runt-related transcription factor 2 (RUNX2) ELISA kit were obtained from Jianglai Biotechnology (China). A live/dead BacLight viability kit was obtained from Invitrogen (USA). Brain heart infusion (BHI) was purchased from OXOID (USA).

### Synthesis of CGP nanoparticles

The synthesis of CGP was carried out in two main steps. Initially, NH_2_-PEG-PCL and 1-amidinopyrazole hydrochloride were mixed in a flask containing 10 mL of dimethyl sulfoxide. To this mixture, N, N-diisopropylethylamine was gradually added under an atmosphere of nitrogen protection, maintaining a molar ratio of 1:1:1. This mixture was then stirred continuously at room temperature for 24 h, followed by dialysis in distilled water for 48 h. The resultant products, G-PEG-PCL, was collected, lyophilized, and stored for further use. The structural confirmation of G-PEG-PCL was performed using a ^1^H nuclear magnetic resonance spectrometer (Bruker AVANCE III HD 600, Germany) operating at 600 MHz. In the second step, Ce6 (1 mg) and G-PEG-PCL (20 mg) were dissolved in 4 mL of dimethyl sulfoxide. This solution was then gradually and dropwise added to 24 mL of ultrapure water, with the mixture stirred in the dark for 24 h. Post-stirring, the samples were collected, dialyzed, lyophilized, and stored for subsequent experiments. For comparison, CPP nanoparticles unmodified with guanidino groups were prepared using NH_2_-PEG-PCL following the procedure described above.

### Physicochemical characterization of CGP

Particle size distribution and zeta potential analyses of free Ce6, blank NH_2_-PEG-PCL nanoparticles (PP), blank G-PEG-PCL nanoparticles (GP), CPP, and CGP were conducted using a Malvern particle size analyzer (Zetasizer Nano ZS-90, Malvern Panalytical). The morphologies of CPP and CGP were visualized through a transmission electron microscope (FEI Talos F200S, Thermo Fisher). The absorption and emission spectra of free Ce6, CPP, and CGP were determined using an ultraviolet‒visible photometer (Ultrospec 7000, Biochrom) and a fluorescence spectrophotometer (F-2710, Hitachi).

### Bacterial association and biofilm penetration of CGP

To quantify the enhanced bacterial association facilitated by the modified guanidino groups in CGP, flow cytometric analysis was conducted. Initially, a bacterial suspension (10^8^ CFU/mL) was prepared by incubating the bacteria in BHI medium on a shaker at 37 ℃ and 200 rpm for 16 h. This was followed by centrifugation (4000 rpm for 6 min) and resuspension in PBS. Subsequently, 500 μL of this bacterial suspension was mixed with 500 μL of CGP (36 μg/mL) and incubated for 5 min. After centrifugation and resuspension in PBS, the samples were analyzed using a flow cytometer (CytoFlex LX, Beckman Coulter). Control samples comprised of PBS, Ce6 (2 μg/mL), and CPP (36 μg/mL).

To assess the biofilm permeation properties of CGP, three-dimensional confocal laser scanning microscope (CLSM, A1, Nikon) was utilized. SYTO-9 (green, λex/λem = 480/500 nm) and Ce6 (red, λex/λem = 400/652 nm) were employed for staining the biofilms and tracking the penetration capability of CGP. Briefly, 50 μL of *E. faecalis* suspension and 450 μL of BHI medium were added to a 24-well plate fitted with round glass coverslips. The plate was then incubated in an anaerobic incubator at 37 °C for 7 days to allow biofilms formation, with medium refreshment every 2 days. After biofilm maturation, the mediums were replaced with CGP (36 μg/mL, 500 μL), and the sample was incubated for 5 min (37 ℃, dark). Subsequently, the biofilms were stained with SYTO-9 dye. After 15 min, the samples were washed and dried naturally away from light. Finally, the biofilms were visualized with CLSM. PBS, Ce6 (2 μg/mL), and CPP (36 μg/mL) were used as control samples.

### ROS and NO generation profiles of CGP

To evaluate NO generation in CGP upon exposure to laser radiation, a modified Griess analysis was employed. In this method, NO rapidly reacts with the Griess reagent to form diazo compounds, which are detectable in the ELISA microplate reader at 540 nm. In a 96-well plate, 50 μL of CGP (54 μg/mL) was mixed with 100 μL of Griess reagent. After 30 min in the dark, the mixtures underwent irradiation with a 660 nm laser (output power of 213 mW/cm^2^) for 0, 1, 3, 5, and 10 min. The absorbance of the samples was then measured at 540 nm using an ELISA microplate reader. For monitoring H_2_O_2_ production during aPDT, the samples were subjected to a similar procedure using an H_2_O_2_ assay kit. Initially, CGP was mixed with the H_2_O_2_ detection reagent and then irradiated as described above. Following this, the mixtures were collected, and their absorbances at 560 nm were measured using an ELISA microplate reader. The production of ^1^O_2_ was detected using SOSG. CGP was first mixed with SOSG and processed as described above; the fluorescence intensity of the mixtures was measured by a multifunctional ELISA microplate reader (λex/λem = 504/525 nm). Control groups for the aforementioned experiments included PBS + Laser, Ce6 + Laser, CPP + Laser, and CGP without laser radiation.

### Imaging of NO, ROS, and H_2_O_2_ production by CGP in *E. faecalis*

To detect NO release from CGP in bacteria, the NO-sensitive fluorescent probe DAF-FM DA was used. Briefly, an *E. faecalis* suspension (10^8^ CFU/mL, 950 μL) and CGP (360 μg/mL, 50 μL) were mixed in an Eppendorf (EP) tube. Subsequently, the EP tube was incubated in an anaerobic incubator for 30 min (37 ℃, dark). DAF-FM DA (5 μM) was then added to the EP tube, followed by irradiation for 5 min. After a 30 min incubation, the sample was washed three times with PBS. Finally, 10 μL of the bacterial suspension was placed onto a slide and photographed using CLSM (λex/λem = 495/515 nm). To assess the ROS production induced by CGP in bacteria, the fluorescent probe DCFH-DA was utilized. Briefly, an *E. faecalis* suspension (950 μL) and CGP (360 μg/mL, 50 μL) were added to an EP tube and incubated in an anaerobic incubator (37 ℃, darkness). After 30 min, the fluorescent probe (5 μM) was added. The samples were processed as described above and photographed using CLSM (λex/λem = 488/525 nm). To investigate the consumption of H_2_O_2_ by CGP in bacteria under aPDT conditions, Amplex red and horseradish peroxidase were employed. First, an *E. faecalis* suspension (950 μL) and CGP (360 μg/mL, 50 μL) were added to an EP tube, which was subsequently incubated in an anaerobic incubator (37 ℃, darkness) for 30 min. Afterward, Amplex red (10 μM) and horseradish peroxidase (10 μM) were added to the EP tube. The samples were processed next as above and photographed using CLSM (λex/λem = 488/525 nm). Control groups for the aforementioned experiments included PBS + Laser, Ce6 + Laser, CPP + Laser, and CGP without laser radiation.

### Antibacterial activity of CGP in vitro

The antimicrobial performance of CGP was evaluated using the colony counting method. Briefly, an *E. faecalis* bacterial suspension (950 μL) and CGP (350 μg/mL, 50 μL) were mixed in an EP tube. After 5 min of incubation in an anaerobic incubator (37 ℃, dark), the sample was irradiated for 5 min and further incubated for 30 min. Subsequently, the bacterial suspension (100 μL) was collected and diluted in a 96-well plate in a gradient (from 10^8^ dilutions to 10^4^ CFU/mL). The sample (5 μL at a time) was spread dropwise in a solid medium, and each concentration was repeated three times before incubation for 24 h in an anaerobic chamber. The number of colonies and bacterial viability were then counted.

To further evaluate the antimicrobial properties of CGP, a live/dead BacLight viability assay was conducted. The bacteria were first treated as described above. After treatment, the bacterial suspension was centrifuged and washed to remove any residues of CGP. The pellet was then resuspended and stained with SYTO-9 and PI for 15 min at room temperature. Then, after another round of centrifuged and washed with PBS to remove excess stain, the bacteria were observed using an inverted fluorescence microscope.

To visualize the morphological changes in bacteria treated with CGP + Laser, scanning electron microscopy (SEM) was utilized. Bacteria were first treated as described above. After treatment, the bacterial cells were centrifuged and washed twice with NS. The bacteria were then fixed using 1 mL of 2.5% glutaraldehyde (4 °C, overnight). The bacteria were subsequently centrifuged and washed three times with NS. After dehydration with graded concentrations (including 30, 50, 70, 90, and 100%) of an ethanol solution, the sample was added dropwise to silicon wafers and allowed to dry naturally. Finally, the bacteria were sputtered with gold and observed with a scanning electron microscope (SU8010, HITACHI). Control groups for these experiments included PBS + Laser, CPP, CGP, Ce6 + Laser, and CPP + Laser.

### Protein leakage and changes in ATP levels in CGP-treated bacteria

To evaluate bacterial protein leakage after treatment with CGP activated by aPDT, the bacteria were processed as previously described. Following the treatment and subsequent centrifugation, the supernatants were collected, and their protein concentration was determined using a BCA protein assay kit. The absorbance at 560 nm was then measured using an ELISA microplate reader. To detect changes in ATP levels within the bacteria, the bacteria were treated as described above, collected by centrifugation, lysed using lysis buffer (200 μL), and centrifuged for 5 min (4 °C, 12,000 *g*). The ATP assay working solution (100 μL) was added to the 96-well plate; subsequently, the sample (20 μL) was added to the plate. Chemiluminescence was detected using the luminometer function of the ELISA microplate reader. Control groups for these experiments included PBS + Laser, CPP, CGP, Ce6 + Laser, and CPP + Laser.

### In vitro biofilm eradication effect of CGP

To evaluate the biofilm eradication efficacy of CGP, a live/dead BacLight viability assay was performed. Initially, a mixture of BHI medium (450 μL) and suspension of *E. faecalis* (50 μL) was added to each well of a 24-well plate containing round glass coverslips. This plate was then incubated in an anaerobic chamber for 7 days to facilitate biofilm formation, with regular medium replacement every 2 days during the incubation period. After aspirating the supernatant, culture medium (250 μL) and CGP (72 μg/mL, 250 μL) were added and coincubated for 5 min. Subsequently, the mixture was irradiated for 5 min. After 30 min of incubation, the biofilm was stained with SYTO-9 and PI dyes for 15 min. Following two washes with PBS, the sample was allowed to dry naturally away from light and CLSM was used to examine the biofilms.

Additionally, to assess the eradication efficacy against *E. faecalis* biofilms, crystalline violet staining was employed. BHI medium (90 μL) and *E. faecalis* suspension (100 μL) were added into a 96-well plate and incubated for 7 days to form biofilms, with the medium refreshed every 2 days. After aspirating the supernatant, medium (50 μL) and CGP (72 μg/mL, 50 μL) were added and coincubated for 5 min, irradiated for 5 min, and further incubated for another 30 min. Subsequent fixation with methanol was followed by staining with crystal violet (0.1%, for 30 min), and the residual biofilms were photographed with a stereomicroscope (SMZ 800N, Nikon). After photographing, the residual biofilm was dissolved with 33% acetic acid and the absorbance at 596 nm was measured using a microplate reader. The biofilm survival rate was calculated as follows: biofilm viability = OD_596_ (sample group)/OD_596_ (PBS group) × 100%. Control groups for the above experiments included PBS + Laser, CGP, Ce6 + Laser, CPP + Laser, and 1% NaClO.

### Biofilm eradication efficacy of CGP in root canals

Single-canal premolar teeth extracted clinically for orthodontic reasons were collected, and the crowns were removed to a distance of 12 mm from the apical foramen in cross-section. The samples were then cleaned and shaped using a ProTaper file (Dentsply Sirona) up to a #40 apical size. Following sterilization, the apical foramen of the teeth was sealed using flowable composite resin (3 M, USA) within a biosafety cabinet. These prepared teeth were then placed in 10 mL EP tubes containing BHI medium (4.95 mL) and an *E. faecalis* suspension (50 μL). The EP tubes were incubated in an anaerobic chamber for 14 days, with the medium refreshed every 2 days. Subsequently, the sample was irrigated with 5 mL of CGP (36 μg/mL) and irradiated for 5 min. To collect residual bacteria, three sterile paper points were then inserted into the root canal for 30 s. These paper points were placed in an EP tube containing 1 mL of NS and stored at − 80 °C for further analysis. After washing and fixation, the roots were dehydrated using graded concentrations (30%, 50%, 70%, 90%, and 100%) of ethanol and then split longitudinally along the long axis. Finally, the biofilms located at the apical regions of the roots were visualized via a scanning electron microscope. On the designated day, the frozen EP tubes were rewarmed at 37 °C. The samples were gradient diluted, and each concentration (100 μL) was spread onto the solid medium. After anaerobic incubation at 37 °C for 24 h, the number of colonies was counted. Control groups included NS, CPP + Laser, and 1% NaClO.

### Biosafety assessment

Erythrocyte aggregation and hemolysis indicate the hemocompatibility of the nanoparticles. Fresh blood from rats was collected and prepared as a 2% suspension of erythrocytes. Briefly, 50 μL of CGP (720 μg/mL) was mixed with 950 μL of the suspension and incubated. Erythrocyte aggregation was assessed through an optical microscope after 30 min (NI–B, Nikon). The hemolysis assay was conducted as follows: 50 μL of CGP (720 μg/mL) was mixed with 950 μL of the suspension and incubated. After 6 h, the mixture was centrifuged and the absorbance of the supernatant at 560 nm was measured. Hemolysis (%) = [OD_560_ (sample) − OD_560_ (PBS)]/[OD_560_ (ddH_2_O) − OD_560_ (PBS)] × 100%. Control samples for these experiments included PBS, Ce6 (40 μg/mL), CPP (720 μg/mL), and 0.25% NaClO (NaClO coincubated with erythrocytes for 2 min).

To further assess the biosafety of CGP, a cytotoxicity assay was employed. The hPDLSCs or MC3T3-E1 cells were cultured in DMEM or MEM-α supplemented with 10% fetal bovine serum and 1% penicillin‒streptomycin at 37 °C (5% CO_2_). The hPDLSCs and MC3T3-E1 cells were seeded into 96-well plates (5000 cells/well) and incubated. After 24 h, the media were replaced with fresh media containing CGP (36 μg/mL) and incubated for another 24 h. The supernatant of each well was aspirated, and fresh media (100 μL) containing Cell Counting Kit-8 reagent (10 μL) was added. The absorbances were measured with an ELISA microplate reader at 450 nm after 4 h of incubation. Control groups included PBS, Ce6 (2 μg/mL), CPP (36 μg/mL), and 0.25% NaClO (coincubation for 1 min).

A calcein/PI cell viability assay was used to further evaluate the cytotoxicity of CGP. Firstly, hPDLSCs were seeded into a 96-well plate (3000 cells/well) and incubated. After 24 h, the mediums were replaced with fresh mediums containing CGP (18 μg/mL) and incubated for an additional 24 h. The supernatant was replaced by calcein/PI solution (100 μL) and incubated in the dark. After 30 min, cell morphology and fluorescence were observed with an inverted fluorescence microscope (Axio Observer 3, ZEISS). Control groups included PBS, Ce6 (2 μg/mL), CPP (36 μg/mL), and 0.25% NaClO (treated for 1 min).

### Osteogenic differentiation in vitro

The osteogenic differentiation capacity of CGP was evaluated using ALP staining and the Alizarin Red S staining method. Initially, MC3T3-E1 cells were seeded into 24-well plates at a density of 25,000 cells/well. After 24 h, the medium was replaced with MEM-α medium containing β-glycerol (10 mM), ascorbic acid (0.05 mM), 10% fetal bovine serum, H_2_O_2_ (40 μM), and CGP (18 μg/mL). The medium was refreshed every 2 days; notably, H_2_O_2_ and CGP were added only on day 0, 4, 8, 12, and 18. After 7 days, the cells were fixed with 4% paraformaldehyde, washed with PBS, and then stained with ALP staining solution for 24 h. Finally, the cells were observed by stereomicroscope and optical microscope. The extent of staining was quantified with ImageJ software. After 21 days of treatment, the cells were fixed and stained with alizarin red working solution for 24 h. MC3T3-E1 cells were subsequently observed in stereomicroscope and optical microscope. Finally, alizarin red was dissolved by using 10% cetylpyridinium chloride, and the absorbance at 596 nm was measured with a microplate reader. Control groups included PBS, H_2_O_2_, and H_2_O_2_ + CPP.

The expression levels of OCN and RUNX2 in MC3T3-E1 cells were examined using ELISA. Mineralization induction treatments were conducted as described above. For OCN measurement, cell supernatants from each group were collected on day 7; and the concentration of OCN was detected by ELISA. For RUNX2 measurement, MC3T3-E1 cells from each group were lysed on day 21, and the samples were centrifuged to collect the supernatants. The total protein concentration of the supernatant was determined with a BCA protein concentration assay kit. Finally, the concentration of RUNX2 was measured using ELISA. Control groups included PBS, H_2_O_2_, and H_2_O_2_ + CPP.

### In vivo treatment of apical periodontitis

Thirty female Sprague‒Dawley rats (6 weeks old) were purchased from Shanghai SLAC Laboratory Animal Co., Ltd. (China). All animals were individually housed within an isolated and sterile confinement under standard conditions. A regular diurnal light cycle of 12 h of light and 12 h of darkness was maintained. After one week of acclimatization period, the rats were randomly assigned to five groups: healthy, PBS, 1% NaClO, CPP + Laser, and CGP + laser. Randomization was performed using a computer-based random order generator. In the experimental protocol for each animal, four researchers were involved in the following capacities: the initial two researchers executed the treatment according to the randomization schema. Exclusively these two researchers had knowledge of the assignment to respective treatment groups. The anesthesia procedure was solely conducted by the third researcher. Ultimately, assessments of bacterial viability and the total volume of resorption cavities were undertaken by the fourth researcher.

The healthy group received no treatment. The remaining groups underwent the following experimental procedures: First, the rats were anesthetized using isoflurane (RWD Life Science, China). Subsequently, the left maxillary first molar of each rat was mechanically ground using a high-speed handpiece without water to access the pulpal cavity. The mesial canals were then shaped using 10–20# K-files (Dentsply Sirona, USA). Following this, the cavities were filled with small cotton balls containing *E. faecalis* suspension and temporarily sealed with glass ionomer cement (GC, Japan).

After 2 weeks, the cements were removed, followed by the irrigation of the root canals using 200 μL of PBS, 1% NaClO, CPP (36 μg/mL), or CGP (36 μg/mL), correspondingly. The treatment groups receiving CPP and CGP were subjected to irradiation employing a 660 nm laser with an output power of 213 mW/cm^2^ for a duration of 5 min. Thereafter, sterile paper points were inserted into the canals to collect residual bacteria, which were then placed into EP tubes containing 1 mL of NS and stored at − 80 °C. Subsequently, the cavities were sealed with flow composite resins. After 3 weeks, the rats were euthanized with an overdose of isoflurane. The left maxillary first molar along with some jawbone specimens was collected. The pulp cavities of the molars were opened and the paper points were inserted into the canals to collect residual bacteria, which were then placed into EP tubes containing NS and stored at − 80 °C. Then, the collected residual bacterial samples were rewarmed and spread onto solid mediums for incubation at the appropriate times. The number of colonies and bacterial survival were calculated. The teeth were fixed using 4% paraformaldehyde. Subsequently, the samples were fixed on the scanning stage of a micro-computed tomography (Skyscan1276, Bruker, Germany) and scanned over 180 degrees at 100 kV and 200 μA with a resolution of 9 μm and a rotation step of 0.2 degrees. Additionally, CTvox and CTan software (by Skyscan) were utilized for 3D reconstruction and analysis of the samples. The samples were then decalcified in a 17% ethylenediaminetetraacetic acid disodium salt solution for 6 weeks. Finally, the samples were fixed, embedded, sectioned, TRAP stained, HE stained, and visualized with an optical microscope.

### Statistical analysis

The results are expressed as the mean ± SEM. The Shapiro‒Wilk test and Brown‒Forsythe test were employed to assess the normality of the data and homogeneity of variance. In instances where the criteria for normal distribution and homogeneity of variance were satisfied, one-way analysis of variance with Tukey’s post hoc analysis was conducted for comparisons among multiple groups. Welch analysis was applied when the data exhibited both normal distribution and uneven variance, and the Tamhane T2 test was utilized for multiple post hoc comparisons. The data were analyzed using Prism 9.0 software (GraphPad, La Jolla, CA). Values of p < 0.05 were deemed significant (*p < 0.05. **p ≤ 0.01, ***p ≤ 0.001, ****p ≤ 0.0001, ns: no significance).

## Results and discussion

### Synthesis and characterization of CGP

To fabricate CGP, the initial step involved the guanidinylation of NH_2_-PEG-PCL using 1-Amidinopyrazole hydrochloride to form G-PEG-PCL [35]. This modified polymer serves as an effective carrier for the photosensitizer Ce6, forming CGP. Notably, G-PEG-PCL enhances the stability of Ce6 in physiological environments, boosts its association with bacteria, aids in biofilm penetration, and additionally functions as a nitric oxide donor. The successful guanidinylation was confirmed by the emergence of of ^1^H nuclear magnetic resonance peak at 3.41 ppm (–N–CH_2_–C–O–). Other characteristic peaks at 3.50 ppm (–CH_2_CH_2_O–) and 2.27 ppm (–O–CO–CH_2_–) were identified, corresponding to the PEG and PCL components of the compound, as depicted in Additional file 1: Fig. S1.

**Fig. 1:**
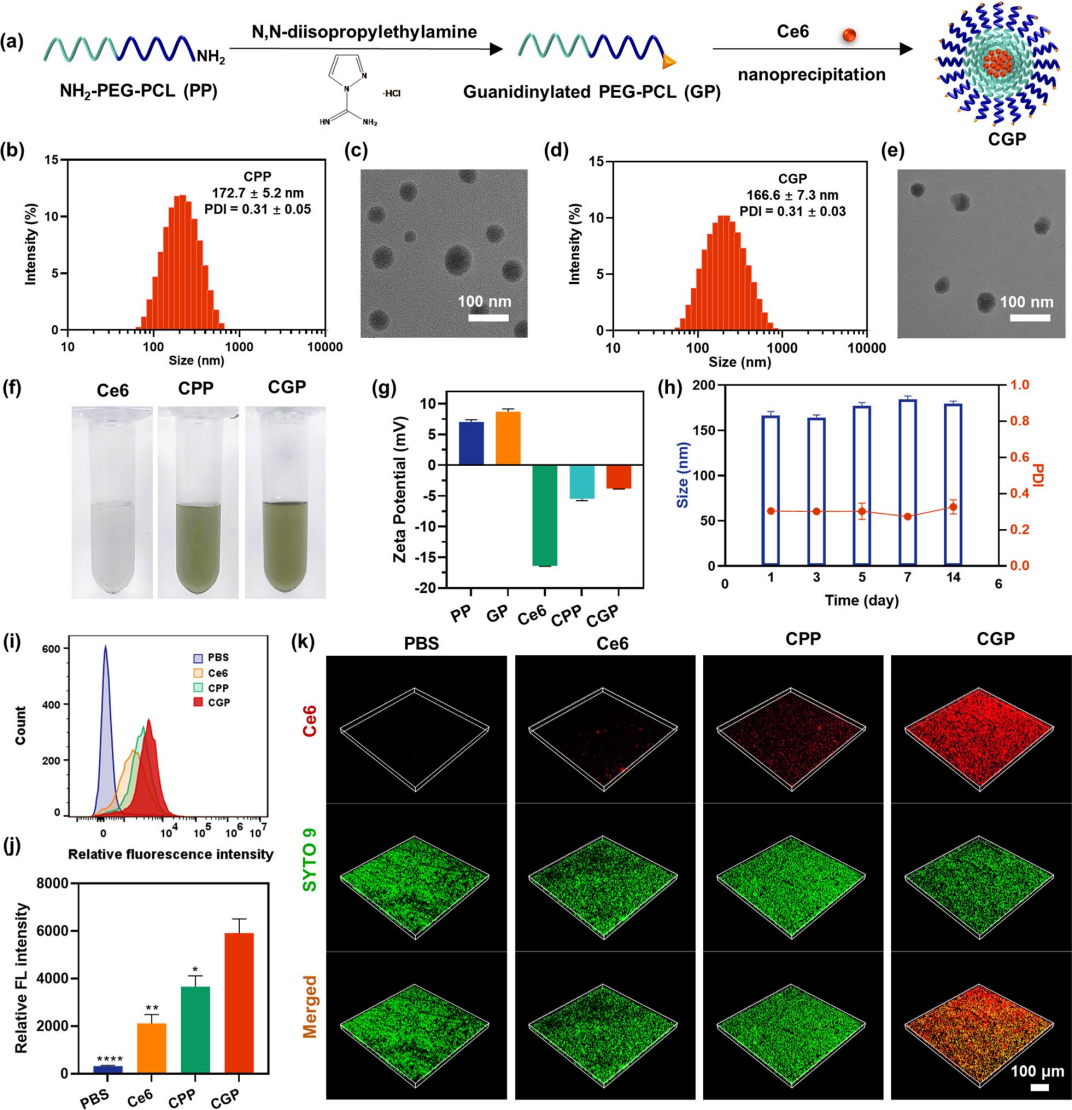
Fabrication and characterization of CGP nanoparticles. **a** Schematic illustration for the preparation of CGP nanoparticles. **b** The particle size distribution and **c** transmission electron microscope images of CPP. **d** The particle size distribution and **e** transmission electron microscope images of CGP. **f** Solubility of Ce6 in different groups. **g** Zeta potential of each group. **h** Stability of CGP during 14 d. **i** Free Ce6, CPP, and CGP internalized by or bound tightly to *E. faecalis* and **j** the quantitative fluorescence intensity of Ce6 in *E. faecalis*. **k** Confocal laser scanning microscope images of *E. faecalis* biofilms treated by various groups. Data are presented as mean ± SEM, n = 3, *p < 0.05, **p ≤ 0.01, ****p ≤ 0.0001. Comparison between CGP versus other groups

Figure 1a illustrates the preparation of CGP nanoparticles. The encapsulation efficiency of Ce6 in CGP was found to be 97.02%, with a loading capacity of 5.57% (Additional file 1: Table S1). As a control, CPP was simultaneously prepared using NH_2_-PEG-PCL loaded with Ce6. The particle sizes and transmission electron microscope images of CPP and CGP exhibited uniform spherical shapes, with sizes of 172.7 ± 5.2 nm and 166.6 ± 7.3 nm (Fig. 1b–e). In aqueous solution, CPP and CGP displayed a distinct green color, contrast to free Ce6, which exhibited poor water solubility, resulting in aggregation at the bottom of the Eppendorf tube (Fig. 1f). These results confirm the successful encapsulation of lipophilic Ce6 by NH_2_-PEG-PCL and G-PEG-PCL, enabling homogeneous dispersion in aqueous solution.

The ζ-potential of PP was 7.04 mV, while that of GP was 8.72 mV (Fig. 1g). The increased ζ-potential in GP relative to PP may be due to the elevated pKa value of the guanidino group, as opposed to the amino group [35, 36]. Furthermore, CGP demonstrated good stability over 14 days at room temperature, as evidenced by the consistent aqueous solution color (Additional file 1: Fig. S2), particle size, and dispersibility index (Fig. 1h). Additionally, the ultraviolet absorption and fluorescence emission spectra of CGP were found to be in close alignment with those of free Ce6 (Additional file 1: Figs. S3 and S4), suggesting that the physical encapsulation of Ce6 did not alter its fundamental absorption and emission characteristics.

### Bacterial association and biofilm permeation of CGP

Flow cytometry analysis was utilized to investigate the impact of guanidino groups on the association of CGP with bacteria. Employing Ce6 as a fluorescent indicator, it was observed that CGP exhibited a notably higher degree of internalization or adhesion to *E. faecalis* bacteria, in comparison to CPP (Fig. 1i). The quantitative fluorescence data further substantiated this observation, revealing that *E. faecalis*s treated with CGP displayed fluorescence intensities approximately 1.61-fold higher than those treated with CPP (Fig. 1j). This finding indicates that guanidino groups in CGP markedly augments its bacterial interaction capabilities [37]. Such an enhancement in bacterial association is pivotal, as it substantially bolsters the bactericidal efficacy of CGP, making it a more potent antimicrobial agent.

The efficacy of antimicrobial agents is significantly influenced by their ability to penetrate biofilms, which are known to hinder the therapeutic effectiveness of such agents [38]. The biofilm permeability of CGP was assessed using a CLSM, employing SYTO-9 as an indicator for biofilm staining. After 5 min of co-incubation with *E. faecalis* biofilm, CLSM analysis showed a faint red fluorescence emanating from Ce6 within the biofilm treated with free Ce6, suggesting limited penetration. This finding underscores the challenge posed by biofilms in obstructing the entry of antibacterial substances. In contrast, a modestly stronger fluorescence signal was observed in the CPP-treated biofilm, implying a slight improvement in biofilm permeability. Remarkably, the CGP-treated biofilm exhibited a significantly intensified fluorescence signal (Fig. 1k). These results suggest that while enhancing Ce6’s solubility and stability, as well as incorporating the amino group in CPP, may slightly improve biofilm permeability, the introduction of guanidinylation markedly enhances CGP's capacity to penetrate biofilms. This enhanced permeability is a key factor in boosting the antimicrobial efficacy of CGP against biofilm-protected bacteria.

### ROS and NO generation driven by aPDT

**Fig. 2:**
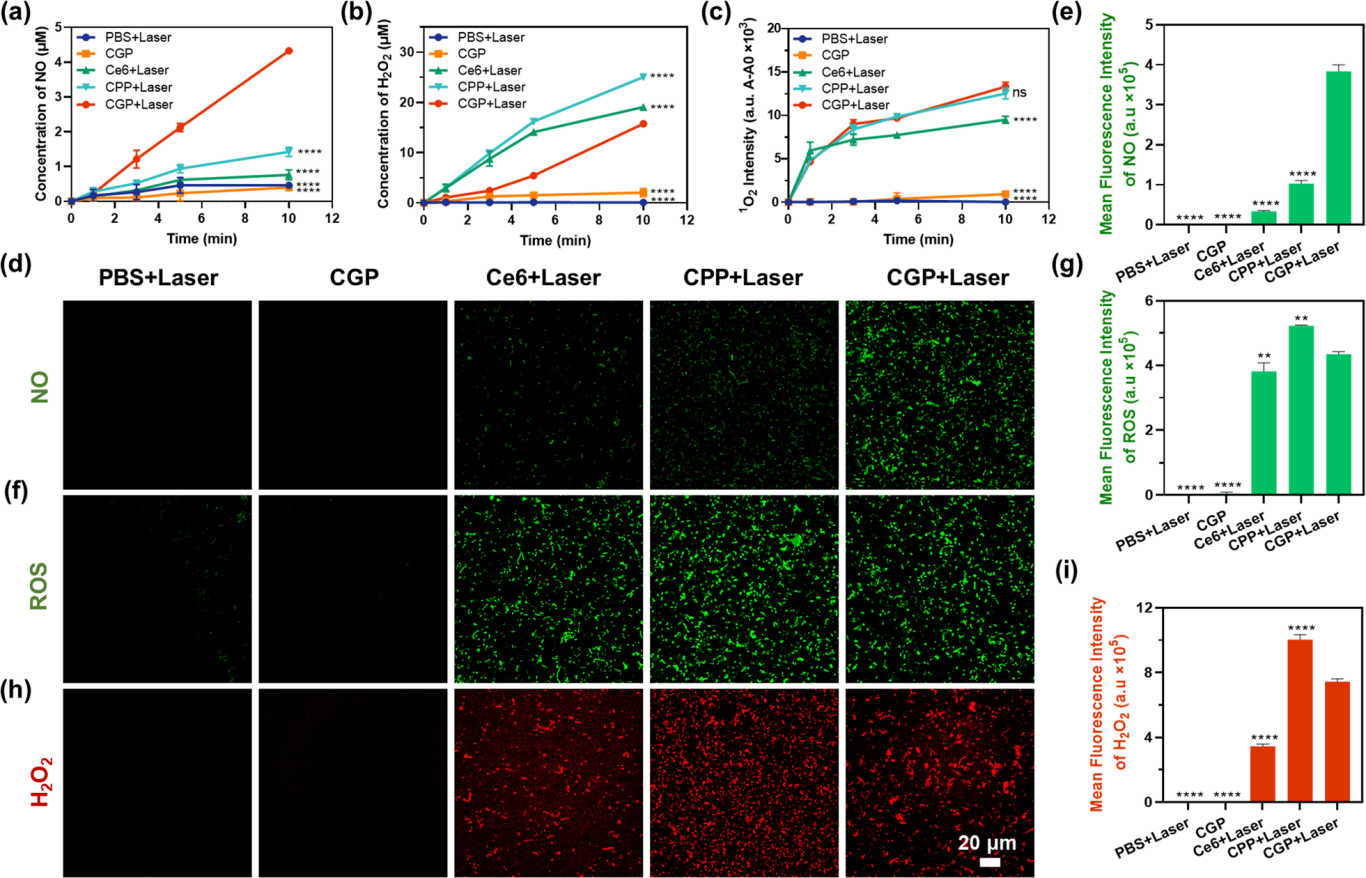
NO and ROS generation profiles of CGP. **a** NO, **b** H_2_O_2_, and **c** ^1^O_2_ generation profiles of each group. **d**, **e** NO, **f**, **g** ROS, and **h**, **i** H_2_O_2_ generation and mean fluorescence intensity in *E. faecalis* bacteria after receiving various treatments. Data are presented as mean ± SEM, n = 3, **p ≤ 0.01, ****p ≤ 0.0001, ns: no significance. Comparison between CGP + Laser versus other groups

After confirming increased bacterial association and improved biofilm penetration by guanidinylated CGP, we employed the Griess assay to evaluate its NO production capabilities in response to aPDT. As shown in Fig. 2a, PBS + Laser, CGP, Ce6 + Laser, and CPP + Laser displayed negligible NO production. In contrast, CGP + Laser exhibited a rapid production of 4.33 μM NO within 10 min. These findings suggest that the capability of CGP nanoparticles to produce NO is likely attributed to the presence of guanidino groups and their activation by aPDT. To further validate the hypothesis that CGP consumes H_2_O_2_ and subsequently produces NO during the aPDT process, we employed an H_2_O_2_ assay kit to detect the generation of H_2_O_2_. The assay operates through the oxidation of divalent iron ions by H_2_O_2_ to produce trivalent iron ions, which combine with xylenol orange to form a purple product [39]. As shown in Fig. 2b, CPP + Laser, and CGP + Laser generated 25.01 and 15.73 μM H_2_O_2_, following irradiation for 10 min. Notably, the H_2_O_2_ generation of CGP + Laser (synchronized with NO generation) was obviously lower than that of CPP + Laser. implying the consumption of H_2_O_2_ in the course of NO generation. This finding suggests that H_2_O_2_ is being consumed during the aPDT process in CGP + Laser group, to facilitate the generation of NO.

It has been reported that ^1^O_2_ also oxidizes the guanidino groups to produce NO [17]. Given that ^1^O_2_ is a pivotal factor in the bactericidal effectiveness of aPDT [40], we anticipated that its production would not be compromised. To investigate this, we employed SOSG to monitor the changes in ^1^O_2_ levels within CGP under aPDT. SOSG possesses faint blue fluorescence but reacts with ^1^O_2_ to produce SOSG endoperoxide, which emits strong green fluorescence [41]. As shown in Fig. 2c, the measured fluorescence intensities of SOSG endoperoxide for both CPP + Laser and CGP + Laser exhibited a similar increase, indicating that CGP can rapidly generate NO by consuming H_2_O_2_-produced during aPDT, without hindering the production of ^1^O_2_. This is crucial for the synergistic antimicrobial and biofilm eradication efficacy of aPDT and NO.

To corroborate the aforementioned results, we employed CLSM to observe the production of NO, ROS, and H_2_O_2_ by CGP in bacteria. The production of NO by CGP in bacteria was detected using DAF-FM DA, a compound that traverses the cell membrane and subsequently undergoes catalysis by intracellular esterase to form DAF-FM, which is incapable of crossing the cell membrane [42]. DAF-FM exhibits weak fluorescence, but it exhibits strong fluorescence upon reacting with NO. As shown in Fig. 2d and e, a notable green fluorescence signal indicative of NO was observed in *E. faecalis* bacteria treated with CGP + Laser, while only negligible fluorescence was noted in bacteria treated with Ce6 + Laser and CPP + Laser. To detect ROS produced by CGP in bacteria, we used DCFH-DA, a non-fluorescent molecule that and can freely traverse the cell membrane and, once inside, is converted into DCFH by intracellular esterase. ROS within the cell can oxidize nonfluorescent DCFH, transforming it into the fluorescent compound DCF [43]. As shown in Fig. 2f and g, the green fluorescence indicative of ROS in *E. faecalis* treated with CPP + Laser was slightly higher than in CGP + Laser treated bacteria, in line with the results obtained in the solution phase (Additional file 1: Fig. S5). H_2_O_2_ produced by CGP in bacteria was detected through the utilization of Amplex Red and horseradish peroxidase [44]. Under the action of horseradish peroxidase, H_2_O_2_ oxidizes Amplex red, resulting in the production of the red fluorescent [45]. Figure 2h and i demonstrate a diminished red fluorescence of H_2_O_2_ in CGP + Laser-treated bacteria in comparison to CPP + Laser. These results further support the possibility that the production of NO actually consumes a part of the ROS and H_2_O_2_ generated during aPDT.

### Antimicrobial effect of CGP in vitro

**Fig. 3:**
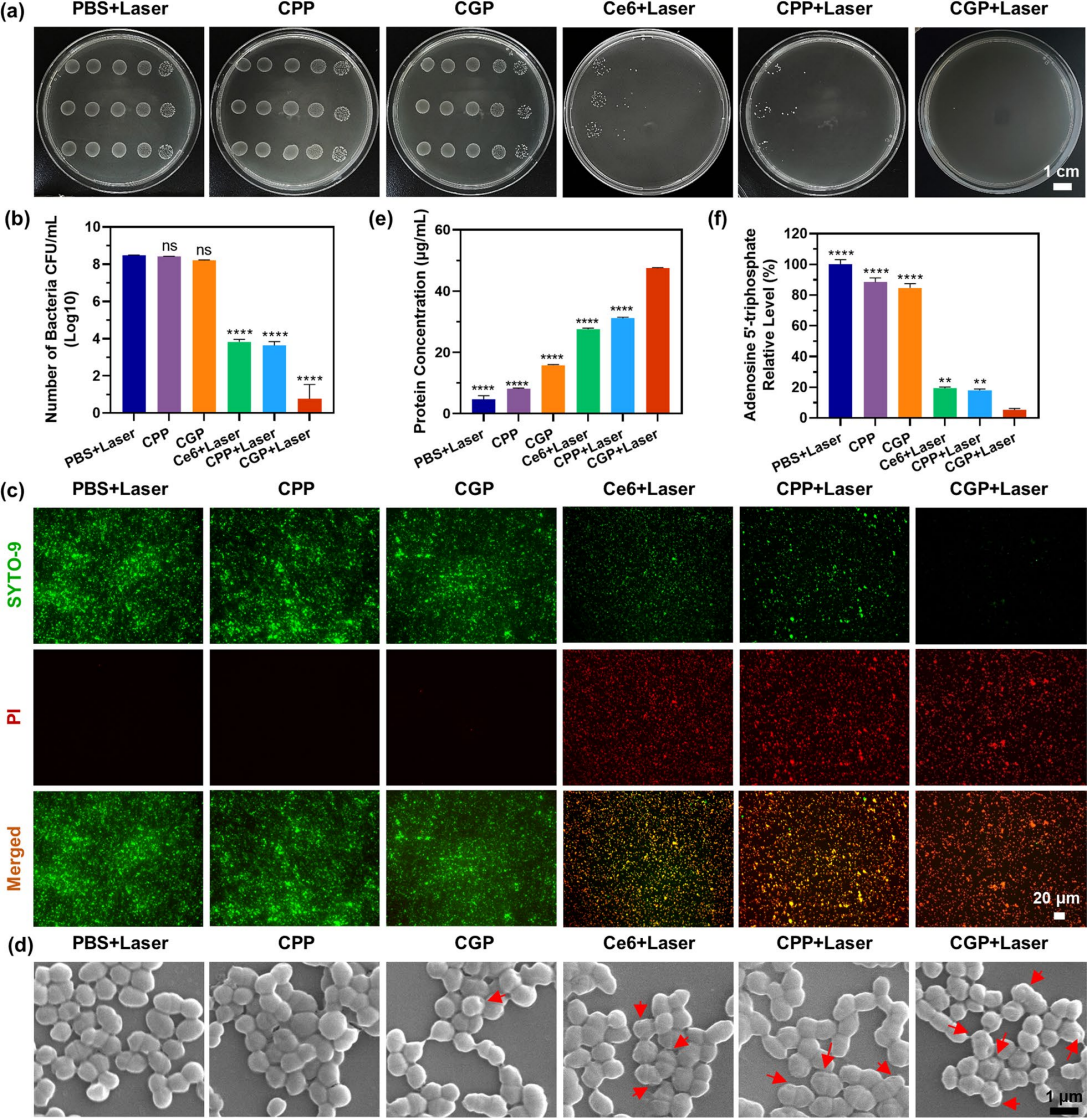
In vitro antibacterial activity of CGP. **a** Representative images of plate samples and **b** bacterial viability, **c** Live/Dead staining, and **d** scanning electron microscope images of *E. faecalis* after receiving various treatments. **e** The amount of total protein released from *E. faecalis* and **f** intracellular adenosine 5'-triphosphate production levels in *E. faecalis* after receiving various treatments. The red arrows point to the holes in cell membranes. The red arrows point to the damaged bacterial membranes. Data are presented as mean ± SEM. In (**b**) n = 3, while in (**e**, **f**) n = 5. **p ≤ 0.01, ****p ≤ 0.0001, ns: no significance. In (**b**) comparison between PBS versus other groups, while in (**e**, **f**) between the CGP + Laser versus other groups

After confirming that the generation of NO driven by aPDT did not impede the generation of ^1^O_2_, the synergistic antimicrobial effects of aPDT and NO were assessed through bacterial colony counting and bacterial live/dead staining. Figure 3a and b displays that the bacterial viability in CPP and CGP without irradiation was higher than 8 log units, which is consistent with that of PBS + Laser. However, a notable reduction in bacterial viability was observed among the remaining groups exposed to laser irradiation: Ce6 + Laser, CPP + Laser, and CGP + Laser, with viability counts of 3.84, 3.63, and 0.77 log units, respectively. This significant decrease in bacterial viability, especially in the CGP + Laser group, highlights the potent antimicrobial effect facilitated by the combination of aPDT and NO. To further elucidate the effects on bacterial cell integrity, we utilized the live/dead BacLight viability kit. Within this kit, the green fluorescent nucleic acid dye SYTO-9 penetrates and labels all bacteria, while PI can only penetrate compromised membranes and the insertion of PI causes a reduction of SYTO 9 fluorescence. As shown in Fig. 3c, PI fluorescence signals were hardly observed in the *E. faecalis* treated with PBS + Laser, CPP, and CGP, suggesting that the bacteria have intact membranes. CPP + Laser and Ce6 + Laser-treated *E. faecalis* exhibited relatively strong green and red fluorescence, indicating a moderate disruption of the bacterial membranes. In contrast, the CGP + Laser-treated bacteria exhibited strong red fluorescence and weak green fluorescence, indicating severe damage to the bacterial membranes. These results confirm that the antimicrobial effect of CGP + Laser is superior to that of CPP + Laser, thus indicating the synergistic antimicrobial effect of aPDT and NO. Furthermore, the study explored the antimicrobial mechanism of the nanoparticles through protein leakage, changes in alterations in ATP levels, and alterations in bacterial morphology.

Figure 3d shows that bacteria treated with PBS + Laser, CPP, and CGP were predominantly smooth and intact, indicating minimal damage. However, the bacteria treated with Ce6 + Laser and CPP + Laser displayed slightly crumpled membranes with small holes, suggesting moderate damage. Notably, bacteria in the CGP + Laser group displayed extensive and deep holes in their cell membranes, indicative of significant damage and disruption. The extent of bacterial membrane damage correlated with the degree of protein leakage observed. As quantified in Fig. 3e. This trend clearly demonstrates the enhanced membrane-disrupting capability of the CGP + Laser treatment. In addition, ATP levels, crucial for cell function, typically diminish in cells undergoing apoptotic, necrotic, or in a toxic state [46]. As shown in Fig. 3f, ATP levels were reduced by 80.61%, 82.05%, and 94.72% in the Ce6 + Laser, CPP + Laser, and CGP + Laser groups, respectively, compared to the PBS group. This substantial reduction in ATP levels, particularly in the CGP + Laser group, underscores the profound impact of the combined aPDT and NO treatment on bacterial vitality. These fundings suggest a synergistic antimicrobial mechanism of aPDT and NO that may be attuned to the following factors: (1) the ability of guanidino groups to permeate biofilms and associate with bacteria, enhancing the overall antimicrobial action; (2) the combined disruptive effect of aPDT-generated ROS and NO on bacterial membranes, leading to significant protein leakage; (3) the synergistic inhibition of mitochondrial ATP production by both ROS and NO, impeding essential bacterial functions; and (4) the triggering of lipid peroxidation, DNA damage, and protein dysfunction by the byproducts of ROS and NO, ultimately resulting in bacterial death. Moreover, unirradiated CPP and CGP were not included in the other experiments as controls due to their negligible antimicrobial activity.

After establishing the synergistic antimicrobial effect of aPDT and NO, we conducted further analysis on the correlation between various concentrations of nanoparticles and their corresponding antimicrobial effectiveness. And compared the antimicrobial efficacy of these nanoparticles with that of NaClO, a commonly used clinical root canal irrigant. As shown in Additional file 1: Figs. S6 and S7, the bactericidal effects of free Ce6 + Laser, CPP + Laser, and CGP + Laser against *E. faecalis* bacteria progressively intensified with increasing nanoparticle concentrations. Additionally, across all tested concentrations, CGP + Laser consistently demonstrated superior antibacterial activity compared to Ce6 + Laser and CPP + Laser. This observation reaffirms the enhanced antimicrobial impact attributed to the synergistic action of aPDT and NO. For instance, bacterial viabilities following treatments with PBS, Ce6 (0.5 µg/mL) + Laser, CPP (8 µg/mL) + Laser, and CGP (8 µg/mL) + Laser were quantified as 9.02, 6.64, 5.14, and 4.30 log units, respectively. Furthermore, NaClO exhibited strong antibacterial properties, with bacterial viability decreasing to 3.28 log units at a 0.5% concentration, and a complete absence of bacterial colonies at a concentration of 1%. Additionally, it is observed that 18 µg/mL of CGP is more effective than 0.5% NaClO, yet not as effective as 1% NaClO. Remarkably, at a concentration of 36 µg/mL, CGP achieved complete eradication of bacterial colonies. These findings indicate that CGP can serve as a formidable alternative to traditional antimicrobial agents in endodontic therapy, offering a novel approach in the fight against bacterial infections in dental treatments.

### Biofilm eradication of CGP in vitro

**Fig. 4:**
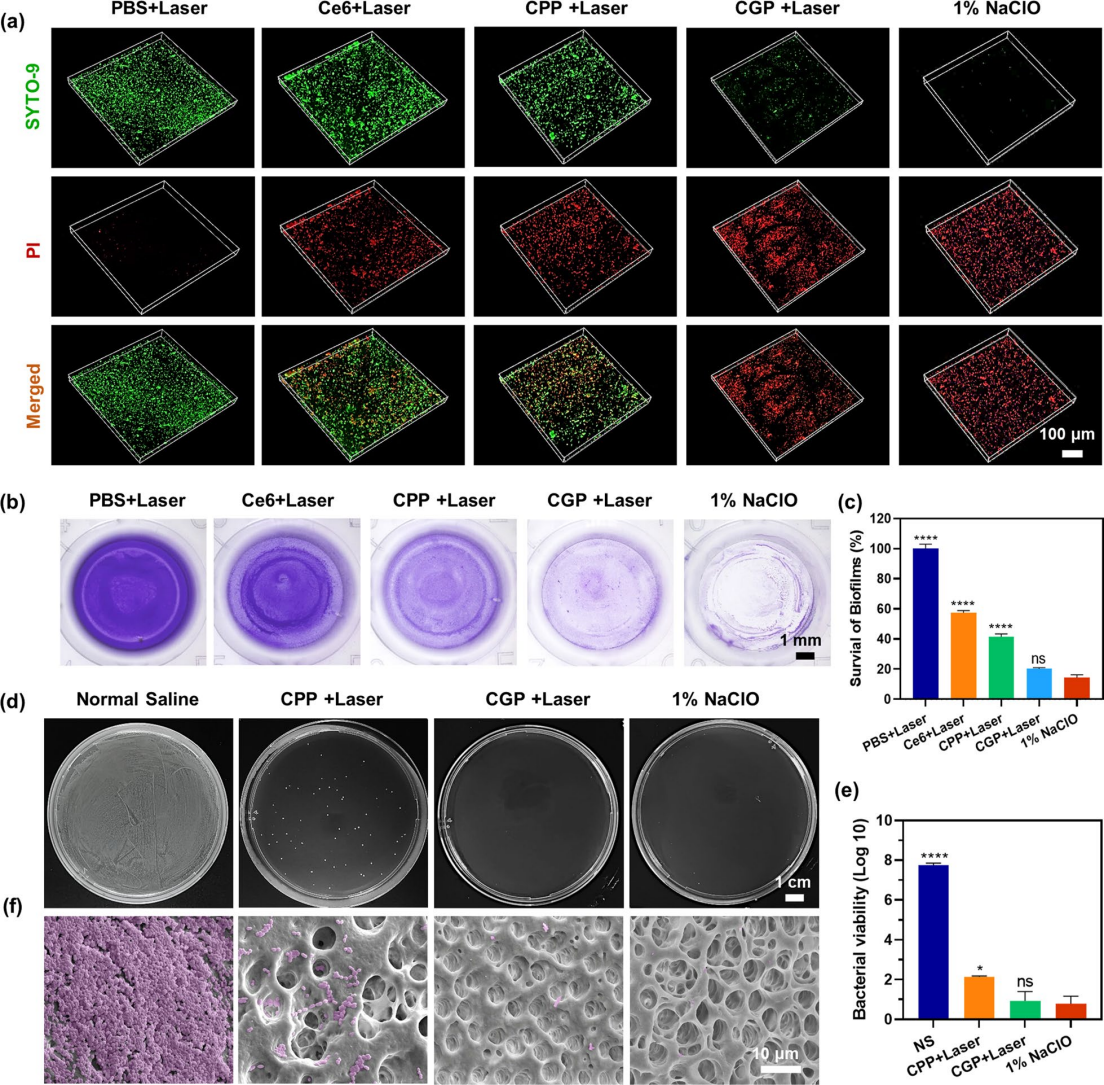
In vitro biofilm eradication efficacy of CGP. **a** Live/Dead staining, **b** crystalline violet staining, and **c** residual rates of *E. faecalis* biofilms after receiving various treatments. **d** Sample plate representative images, **e** bacterial viability, and **f** scanning electron microscope images of *E. faecalis* biofilms in human extracted teeth after receiving various irrigations. Data are presented as mean ± SEM, n = 3, *p ≤ 0.05, ****p ≤ 0.0001, ns: no significance. Comparison between 1% NaClO versus other groups

Considering the remarkable abilities of CGP in terms of biofilm penetration and synergistic antimicrobial activity, we postulated that it could efficiently eradicate biofilms. Our initial investigation into this matter involved assessing the biofilm eradication property of CGP on *E. faecalis* biofilms, utilizing the live/dead bacterial staining method. As shown in Fig. 4a, the presence of green fluorescence from SYTO-9 in the *E. faecalis* biofilm treated with PBS + Laser was evident, whereas the red fluorescence from PI was scarcely observed. Comparatively strong signals of both green and red fluorescence were visualized in the biofilms treated with Ce6 + Laser and CPP + Laser, indicating that only a portion of the biofilms was disrupted. In contrast, intense PI signals were observed in the biofilms treated with CGP + Laser and 1% NaClO, implying that the biofilms were nearly eradicated. To corroborate these findings, we also utilized crystal violet staining to evaluate the biofilm eradication efficacy of CGP. As shown in Fig. 4b and c, the biofilms remained relatively intact following treatment with Ce6 + Laser and CPP + Laser. In stark contrast, CGP + Laser and 1% NaClO exhibited efficient eradication efficacy, leaving only 20.24% and 14.22% of biofilm residuals. On the one hand, these results further indicate that CGP exhibits superior synergistic biofilm eradication properties compared to aPDT alone. On the other hand, although NaClO, due to its corrosive nature, slightly surpasses CGP in biofilm eradication efficacy, its potential for severe side effects and well-known cytotoxicity must be acknowledged. Consequently, we believe that CGP still holds potential for application in root canal irrigation. In addition, it was evident that the antimicrobial and biofilm eradication efficacies of free Ce6 + Laser were relatively inferior compared to those of CPP + Laser and CGP + Laser. Therefore, in subsequent experiments, free Ce6 + Laser was excluded from being used as a control group, allowing for a more focused analysis on the potent biofilm-targeting properties of CGP.

Encouraged by the favorable outcomes of CGP + Laser in live/dead bacterial staining and crystal violet staining, we proceeded to cultivate *E. faecalis* biofilms in the root canals of extracted human teeth to assess the biofilm eradication efficacy. As shown in Fig. 4d and e, the bacterial viability post CPP + Laser treatment was observed at 2.14 log units, whereas CGP + Laser treatment reduced it to 0.93 log units. Notably, the efficacy of CGP + Laser was nearly akin to that of 1% NaClO, which showcased a bacterial viability of just 0.77 log units. To gain further insights, the treated tooth was longitudinally dissected along its long axis, focusing on the apical portion of the root canals for detailed observation under a scanning electron microscope. As shown in Fig. 4f, abundant *E. faecalis* biofilm densely colonizing the root canal wall and obstructing the dentinal tubules in the control group treated with 0.9% NS. In contrast, only a few bacterial residues were evident in CPP + Laser treated canals, while the canals treated with CGP + Laser and 1% NaClO appeared virtually devoid of any bacterial presence. These results collectively suggest that CGP + Laser treatment is highly effective in eradicating *E. faecalis* biofilm within the root canals, demonstrating an efficacy nearly comparable to the commonly used endodontic irrigant, 1% NaClO. Given the temporal and spatial controllability, along with its similar antimicrobial properties and biofilm eradication efficacy to NaClO, CGP emerges as a potential candidate for clinical applications in endodontics.

### Hemocompatibility and biocompatibility assays

**Fig. 5:**
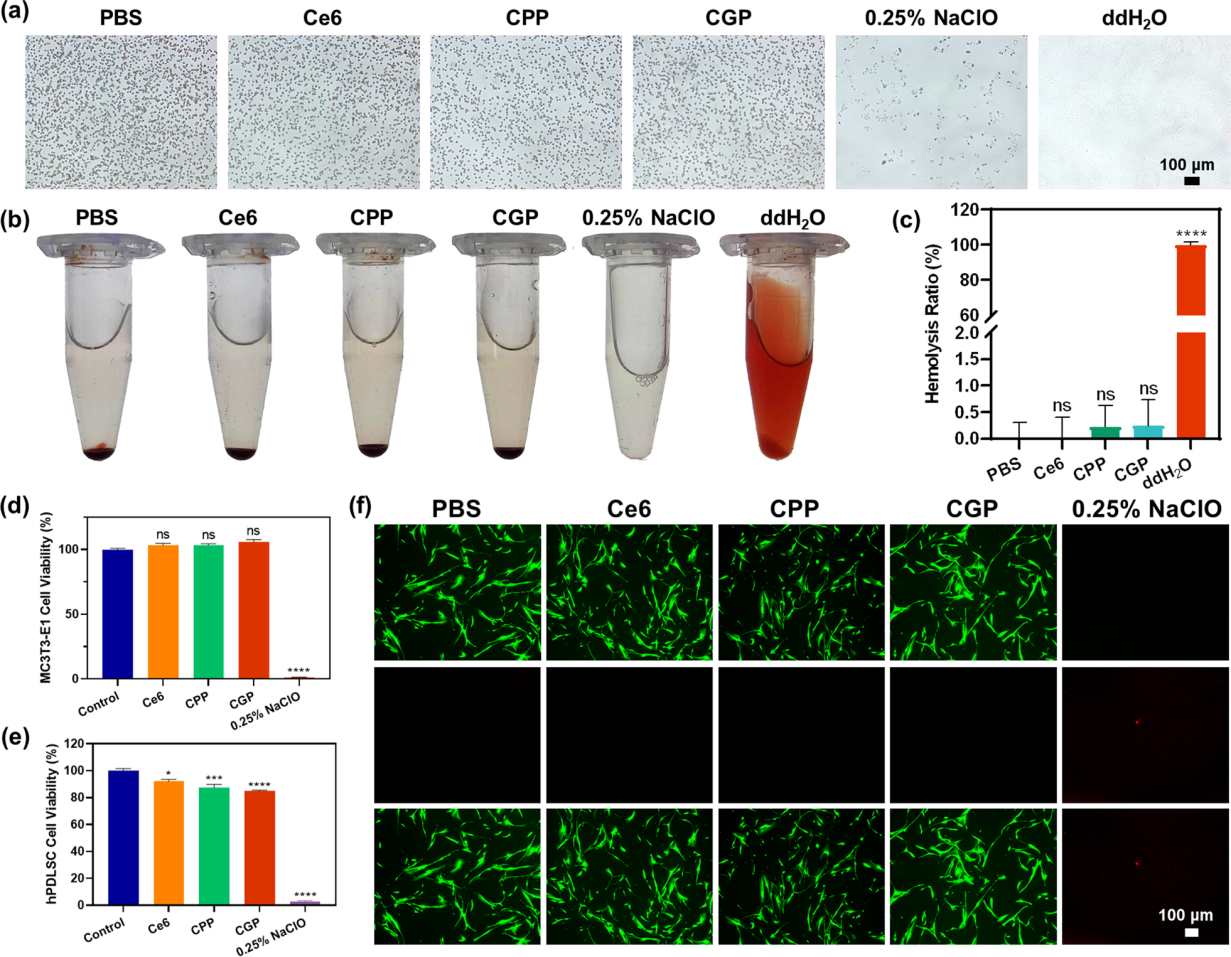
Blood compatibility and biocompatibility of CGP. **a** Microscopic images of erythrocyte aggregation in different treatment groups. **b**, **c** Hemolytic effects in different treatment groups. **d** Cell Counting Kit-8 assay of MC3T3-E1 and **e** hPDLSCs cells after treated with various groups. **f** Live/dead cell staining in different groups of hPDLSCs. Data are presented as mean ± SEM. In (**c**) n = 3, while in (**d**, **e**) n = 5. *p < 0.05, ***p ≤ 0.001, ****p ≤ 0.0001, ns: no significance. In (**c**) comparison between PBS versus other groups, while in (**d**, **e**) between the control group versus other groups

Biosafety is a paramount consideration for in vivo applications. In this context, we used erythrocyte aggregation assay and hemolysis assay to demonstrate the blood compatibility of the nanoparticles. As shown in Fig. 5a and b, when tested with free Ce6 (2 μg/mL), CPP (36 μg/mL), and CGP (36 μg/mL), there were no noticeable enhancements in erythrocyte aggregation, nor were there any disruptions in erythrocyte morphology. Moreover, in the absence of laser irradiation, free Ce6, CPP, and CGP exhibited < 0.25% erythrocyte hemolysis (Fig. 5c). This result starkly contrasts with the outcome of erythrocytes treated with 0.25% NaClO, which lost their integrity, leaving only cellular fragments observable under the microscope. Due to the strong corrosive and oxidizing properties of NaClO [47], the blood was decolored after co-incubation with 0.25% NaClO for 1 min.

Besides, the cell viability of CGP was consistently > 85% after co-incubation with MC3T3-E1 cells or hPDLSCs for 24 h (Fig. 5d and e). Notably, the morphology of hPDLSCs remained unaffected after this period, with no significant red fluorescence from PI staining observed (Fig. 5f). As predicted, NaClO exhibited pronounced cytotoxicity, with less than 2.5% cell survival after 1 min of co-incubation with hPDLSCs and MC3T3-E1 cells. The above experiments show that CGP has good hemocompatibility and biocompatibility, with great potential for clinical applications.

### Osteogenic differentiation in vitro

**Fig. 6:**
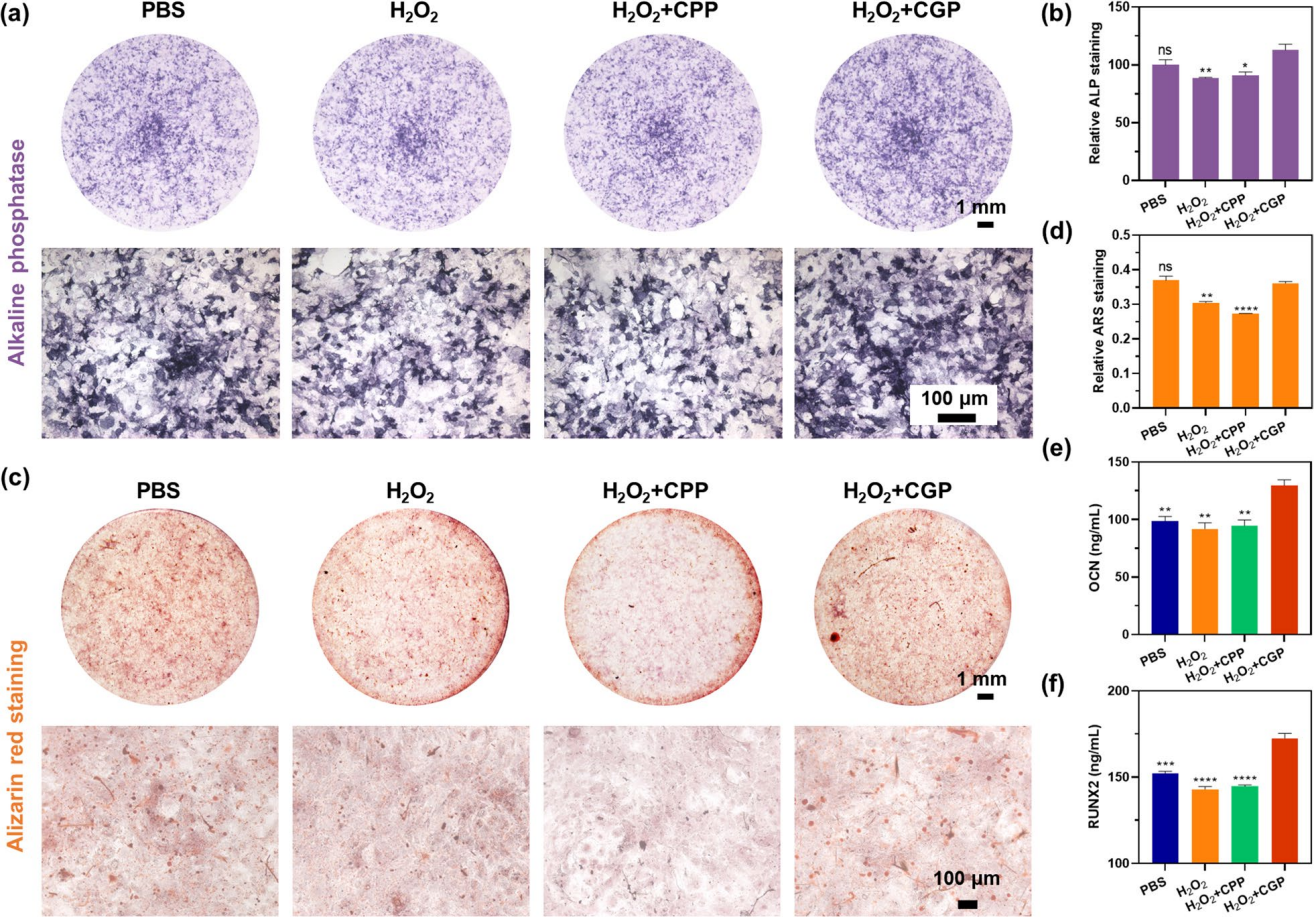
Mechanisms of CGP promoting repair of periapical bone defects. **a**, **b** Images and quantitative data of ALP staining and **c**, **d** alizarin red staining in MC3T3-E1 cells after receiving various treatments in mineralizing conditions. **e** The expression levels of OCN and **f** RUNX2 in MC3T3-E1 cells after receiving various treatments in mineralizing conditions. Data are presented as mean ± SEM, n = 3, *p < 0.05, **p ≤ 0.01, ***p ≤ 0.001, ****p ≤ 0.0001, ns: no significance. Comparison between H_2_O_2_ + CGP versus other groups

In the treatment of AP, in addition to controlling the infection, we hope that the CGP will also promote periapical bone repair and regeneration. Bone regeneration is a complex process associated with inflammation, angiogenesis, and osteogenesis. NO may play an important role in ALP activity and mineralization in osteoblastic lineage [33]. Our cellular experiments employed H_2_O_2_ to simulate inflammatory environments, and drive CGP to generate NO. After 7 days of treating MC3T3-E1 cells with H_2_O_2_ and H_2_O_2_ + CPP under mineralized conditions (add β-glycerol and ascorbic acid in the medium), the degree of ALP staining was slightly lower than that of PBS, while H_2_O_2_ + CGP was darker than PBS (Fig. 6a). This was corroborated by quantitative ALP data, which revealed that the ALP activity in the H_2_O_2_ + CGP group was 19% higher compared to H_2_O_2_ alone (Fig. 6b). During osteoblast differentiation, calcium compounds are deposited on the cell surface to form calcium nodules. These calcium nodules can be stained orange by using alizarin red staining [48]. After 21 days of treatment, MC3T3-E1 cells treated with H_2_O_2_ and H_2_O_2_ + CPP showed fewer mineralized nodules than those treated with PBS, whereas they were more abundant in H_2_O_2_ + CGP (Fig. 6c and d). These findings suggest that while H_2_O_2_ and H_2_O_2_ + CPP substantially impeded the mineralization process of MC3T3-E1 cells, H_2_O_2_ + CGP alleviated the adverse impacts of H_2_O_2_ and reinstated the count of mineralized nodules to levels comparable to those of the PBS group.

ELISA was utilized to analyze the secretion of osteogenesis-related markers, encompassing OCN and RUNX2, in MC3T3-E1 cells with various treatment. OCN is known to play a crucial role in bone metabolism, particularly in calcium homeostasis and bone mineralization processes [49]. RUNX2, on the other hand, functions in the growth and differentiation of osteoblasts by stimulating the expression of subsequent biomarkers [50, 51]. As shown in Fig. 6e, the concentration of OCN in the supernatants of MC3T3-E1 cells varied across treatment groups. The cells treated with PBS showed an OCN level of 98.6 ng/mL. In the presence of H_2_O_2_ and H_2_O_2_ + CPP, this level decreased slightly to 91.9 and 94.7 ng/mL, suggesting some disruption of osteogenic activity under inflammatory conditions. Remarkably, cells treated with H_2_O_2_ + CGP demonstrated a significant increase in OCN concentration, reaching 129.6 ng/mL. This suggests that the presence of CGP, not only mitigates the inhibitory effect of H_2_O_2_ but also enhances osteogenic activity. Similarly, the RUNX2 levels in MC3T3-E1 cell lysates exhibited a corresponding pattern. Cells treated with PBS had a RUNX2 level of 152.0 ng/mL. In the presence of H_2_O_2_ and H_2_O_2_ + CPP this level decreased slightly to 143.0 and 144.6 ng/mL. However, in the H_2_O_2_ + CGP group, there was a notable increase in RUNX2 concentration, reaching 172.6 ng/mL (Fig. 6f). This increase suggests that CGP enhances osteoblast differentiation and bone formation, even under conditions of inflammatory.

These results indicate a notable suppression of osteogenic differentiation in the presence of H_2_O_2_, a ROS that is commonly present in inflammatory conditions [52]. However, the introduction of CGP appears to counteract this inhibitory effect. The mechanism underlying this phenomenon involves the consumption of H_2_O_2_ by CGP, leading to the generation of NO. This release of NO from CGP is instrumental in stimulating osteogenic differentiation in MC3T3 cells, as evidenced by increased ALP activity at early stages of differentiation, initiation of calcium deposition at later stages, and the up-regulation of key osteogenic markers such as OCN and RUNX2. It is noteworthy that this assay does not accurately mirror the actual in vivo scenario, but H_2_O_2_ is a ROS that persistently exists in inflammatory sites in the long run, and ROS produced by periapical tissues obstruct osteoblast differentiation [53]. In therapeutic applications, residual H_2_O_2_, either from aPDT or periapical inflammation, could potentially oxidize CGP, thereby producing trace amounts of NO. This process is expected to promote the repair and regeneration of periapical bone defects, highlighting the potential of CGP as a therapeutic agent in conditions where inflammation and oxidative stress impede normal bone healing and regeneration processes.

### In vivo treatment of apical periodontitis

**Fig. 7:**
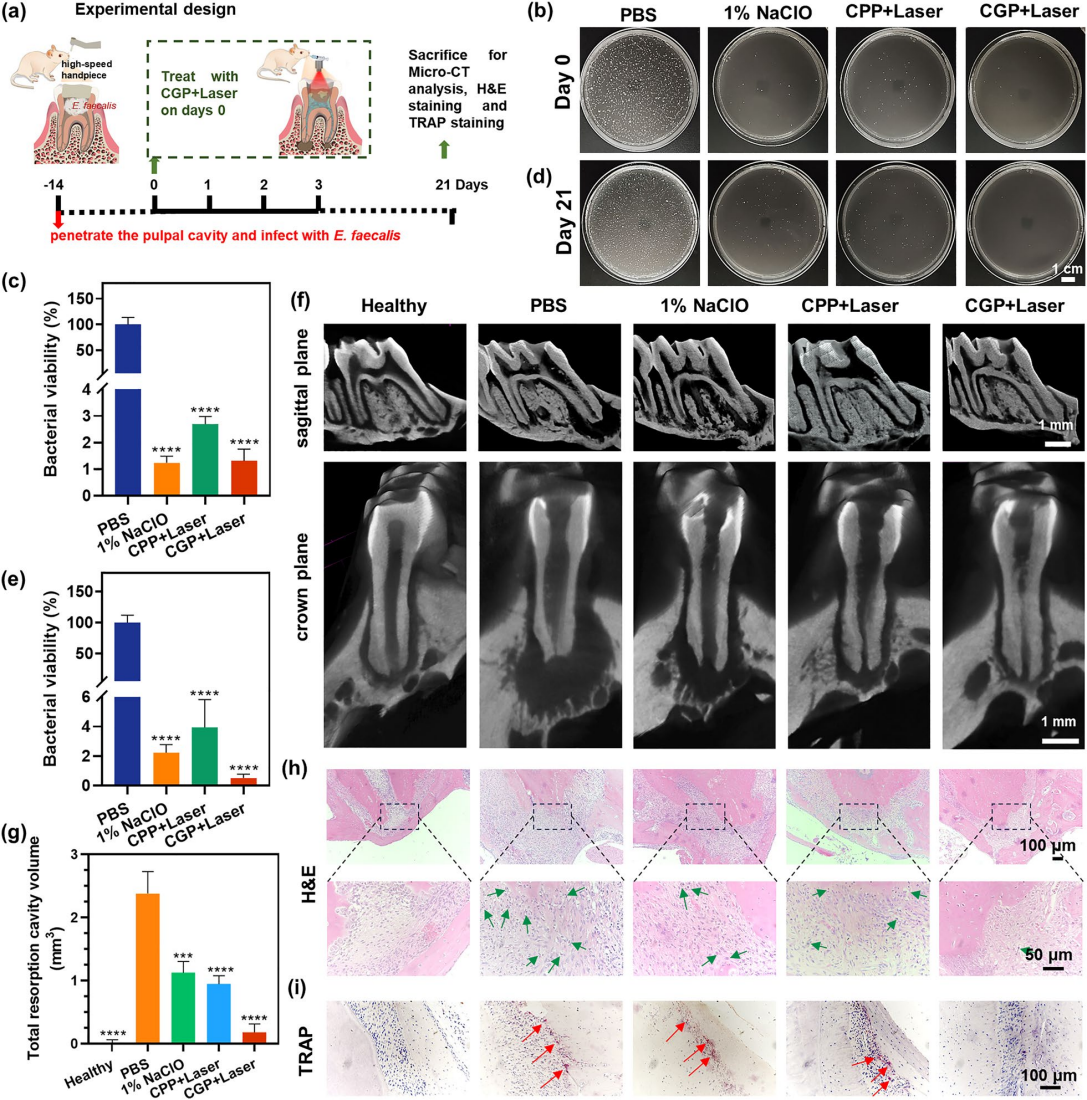
In vivo therapeutic efficacy of CGP apical periodontitis model rats. **a** Experimental design of apical periodontitis model rats. **b**, **c** Sample plate representative images and bacterial viability of *E. faecalis* in rat teeth after receiving various irrigations on day 0 and **d**, **e** day 21. **f** Representative images and **g** the total resorption volume of the left maxillary first molar in each group. **h** Representative HE staining and **i** TRAP staining. The green arrows point to the neutrophils and the red arrows point to active osteoclasts. Data are presented as mean ± SEM, n = 6, ****p ≤ 0.0001. Comparison between PBS versus other groups

CGP exhibited favorable biocompatibility, antimicrobial ability, eradication of biofilm, and promotion of mineralization properties in vitro experiments. This provides a foundation for carrying out in vivo therapeutic evaluation using a rat apical periapical model. Figure 7a presents the detailed experimental procedure for establishing an AP model for treatment and effect assessment. Briefly, the pulp cavity of the left maxillary first molar of the rat was opened, and a small cotton ball containing *E. faecalis* suspension was placed to infect the root canal, after which the crown was sealed with glass ions. Following 2 weeks of infection, the rats were treated with PBS, 1% NaClO, CPP + Laser, and CGP + Laser, respectively. After 3 weeks of treatment, the left maxillary first molar and the surrounding maxillary area were collected from the rats, and the extent of periapical bone defects was measured using micro-computed tomography. The results revealed notable reductions in bacterial viability across all treatment groups on the day of irrigation (Fig. 7b and c) and at day 21, indicating significant antimicrobial effects (Fig. 7d and e). Consistent with the results of root canal irrigation in extracted teeth, the antimicrobial effect was higher than 96% in all groups. Figure 7f shows the coronal and sagittal planes of the three-dimensional reconstructed images of the representative first molar for each group. The figure clearly illustrates large bone resorption cavity in the periapical tissues of the PBS group, indicating that *E. faecalis* infection of the root canal led to substantial periapical bone defect. In contrast, this defect obviously decreased in the 1% NaClO, CPP + Laser, and CGP + Laser groups, indicating gradual healing of the bone defect after successful infection control. Particularly noteworthy is the significantly smaller resorption cavity observed in the CGP + Laser group compared to the other groups, with its periapical condition closely resembling that of the healthy group, suggesting enhanced healing of periapical bone defects. And the quantitative data presented in Fig. 7g, the volume of the resorption cavity was 2.38, 1.12, 0.94, and 0.18 mm^2^ for PBS, 1% NaClO, CPP + Laser, and CGP + Laser, respectively. CPP + Laser exhibited a 7.5% reduction in bone defects in periapical inflammation treatment compared to the NaClO group (PBS group as 100%), likely due to the extrusion effect of NaClO, causing periapical tissue toxicity [54]. The conventional syringe irrigation method was employed in this study, and apical extrusion was inevitable except for negative pressure irrigation systems [55]. Remarkably, the resorption cavity of CGP + Laser was 32.2% lower than that of CPP + Laser. CGP + Laser showed excellent therapeutic effects with antimicrobial effects, and the production of NO during therapy aided in the healing of periapical bone defects. The synergistic application of aPDT with NO has been proven to treat AP both effectively and safely.

H&E and TRAP staining were performed to facilitate additional evaluation of the synergistic impact of aPDT and NO. As shown in Fig. 7h, the periapical area of the PBS group exhibited abundant neutrophil infiltration, whereas the 1% NaClO and CPP + Laser groups displayed minor inflammatory cell infiltration, indicates that inflammation in the periapical tissues gradually diminished after successful infection control. Conversely, the CGP + Laser group showed minimal neutrophil infiltration, suggesting a milder inflammatory response in this group. Additionally, the periapical tissues in this group exhibited morphological integrity without discernible defects, indicating the most favorable treatment outcome. As shown in Fig. 7i, TRAP staining showed numerous active osteoclasts in the periapical tissues of the PBS, while the number of osteoclasts was reduced in NaClO and CPP + Laser group. Notably, osteoclasts were virtually absent in CGP + Laser, while histological features and the periodontal ligament space were similar to those of healthy group. These findings may be attributed to the potent antimicrobial effect and the capacity to stimulate bone regeneration associated with CGP + Laser, demonstrating optimal therapeutic efficacy in endodontic treatment. In addition, no significant toxicity of CGP was observed during treatment, and no pathological abnormalities were observed in the major organs of the rats (Additional file 1: Fig. S8), which indicated that the safety of our multifunctional antibacterial nanoplatform [56, 57]. These findings substantiate the potential future application of CGP + Laser in endodontics, while also presenting a novel candidate treatment strategy for root canal irrigation.

Although this study has yielded some promising results, it is important to acknowledge certain limitations. Subsequent research should focus on conducting comprehensive analyses of the exact mechanisms and efficacy of CGP within multispecies biofilms and intricate root canal structures, such as C-shaped, highly curved, and accessory canals [58]. While specific concentrations of CGP + Laser demonstrate comparable antimicrobial properties, NaClO still maintains a unique position in root canal irrigation owing to its potent antimicrobial and tissue-dissolving characteristics. And we believe that CGP + Laser can serve as a compelling alternative in certain cases. Furthermore, it may hold promise in periodontal therapy, managing other biofilm infections, and various other applications.

## Conclusions

This study demonstrated a versatile aPDT-driven controlled NO generation system, denoted as CGP, which was employed for synergistic aPDT/NO treatment of AP. During the root-canal irrigation process, the guanidino groups on the surface of CGP successfully penetrated the biofilms. These Guanidino groups can be oxidized by H_2_O_2_ generated during aPDT without impacting ^1^O_2_ generation. The combination of NO and aPDT exhibited a noteworthy synergistic antimicrobial effect and a remarkable ability to eradicate biofilms, both in vitro and in vivo. Following the successful elimination of root canal biofilms, the produced NO promoted the repair of bone defects in periapical tissues, thereby significantly facilitating the healing of AP. Consequently, an effective root canal irrigation system was developed.

### Supplementary Information

**Additional file 1: Table S1.** Ce6 loading capacity and encapsulation efficiency of the CGP. **Fig. S1.** ^1^H nuclear magnetic resonance spectra of G-PEG-PCL. **Fig. S2.** The stability of CGP during 14 days. **Fig. S3.** Ultraviolet-visible absorption spectra of free Ce6, CPP, and CGP. **Fig. S4.** Fluorescence emission spectra of free Ce6, CP, and CGP. **Fig. S5.** Total ROS generation profiles of different groups over various durations. Data are presented as mean ± SEM, n = 3, **** p ≤ 0.0001. Comparison between CGP+Laser versus other groups. **Fig. S6.** Representative images of plate samples of *Enterococcus faecalis* after various treatments. **Fig. S7.** bacterial viability of *Enterococcus faecalis* after various treatments. **Fig. S8.** HE staining of heart, lung, liver, spleen, and kidney in healthy group and CGP+Laser treated AP group.

## Data Availability

The datasets used and analyzed during the current study are available from the corresponding author on reasonable request.

## Abbreviations

AP: Apical periodontitis
NaClO: Sodium hypochlorite
aPDT: Antimicrobial photodynamic therapy
ROS: Reactive oxygen species
H_2_O_2_: Hydrogen peroxide
^1^O_2_: Singlet oxygen
NO: Nitric oxide
G-PEG-PCL: Guanidinylated poly (ethylene glycol)-poly(ε-caprolactone)
NH_2_-PEG-PCL: Amino-terminated poly (ethylene glycol)-poly(ε-caprolactone)
Ce6: Chlorin e6
CGP: G-PEG-PCL loaded with Ce6
CPP: NH_2_-PEG-PCL loaded with Ce6
PP: Blank NH_2_-PEG-PCL nanoparticles
GP: Blank G-PEG-PCL nanoparticles
*E. faecalis*: *Enterococcus faecalis*
PBS: Phosphate-buffered saline
NS: Normal saline
TRAP: Tartrate-resistant acid phosphatase
HE: Hematoxylin-eosin
SOSG: Singlet oxygen sensor green
DCFH-DA: Dichlorodihydrofluorescein diacetate
DAF-FM DA: 3-Amino,4-aminomethyl-2',7'-difluorescein, diacetate
ATP: Alterations in adenosine 5'-triphosphate
hPDLSCs: Human periodontal ligament stem cells
ALP: Alkaline phosphatase
ELISA: Enzyme-linked immunosorbent assay
OCN: Osteocalcin
RUNX2: Runt-related transcription factor 2
CLSM: Confocal laser scanning microscope
