# Supplementary material for: Photodynamic and nitric oxide therapy-based synergistic antimicrobial nanoplatform: an advanced root canal irrigation system for endodontic bacterial infections
Source: J Nanobiotechnology. 2024 Apr 30;22:213. doi: 10.1186/s12951-024-02483-8 (PMC11059741; doi:10.1186/s12951-024-02483-8)
Supplement: Supplementary file 1 — Additional file 1: Table S1. Ce6 loading capacity and encapsulation efficiency of the CGP. Fig. S1. 1H nuclear magnetic resonance spectra of G-PEG-PCL. Fig. S2. The stability of CGP during 14 days. Fig. S3. Ultraviolet-visible absorption spectra of free Ce6, CPP, and CGP. Fig. S4. Fluorescence emission spectra of free Ce6, CP, and CGP. Fig. S5. Total ROS generation profiles of different groups over various durations. Data are presented as mean ± SEM, n = 3, **** p ≤ 0.0001. Comparison between CGP+Laser versus other groups. Fig. S6. Representative images of plate samples of Enterococcus faecalis after various treatments. Fig. S7. bacterial viability of Enterococcus faecalis after various treatments. Fig. S8. HE staining of heart, lung, liver, spleen, and kidney in healthy group and CGP+Laser treated AP group. [file 12951_2024_2483_MOESM1_ESM.docx]

**Photodynamic and Nitric Oxide Therapy-based Synergistic Antimicrobial Nanoplatform: An Advanced Root Canal Irrigation System for Endodontic Bacterial Infections**

*Youyun Zeng^#^, Xiangyu Hu^#^, Zhibin Cai, Dongchao Qiu, Ying Ran, Yiqin Ding, Jiayi Shi, Xiaojun Cai*, Yihuai Pan**

School and Hospital of Stomatology, Wenzhou Medical University, Wenzhou 325027, China

**# These authors contributed equally to this research.**

***** **Corresponding authors:**

Xiaojun Cai, email: cxj520118@njtech.edu.cn, cxj520118@wmu.edu.cn.
Yihuai Pan, email: yihuaipan@wmu.edu.cn.

**Additional file**

**Keywords:** antimicrobial photodynamic therapy, nitric oxide gas therapy, root canal irrigation, multifunctional nanoparticles, biofilm, antimicrobials, osteogenesis

**Table S1** Ce6 loading capacity and encapsulation efficiency of the CGP.

| Experiment | Total Ce6 (mg) | Total G-PEG-PCL (mg) | Ce6 loaded in  G-PEG-PCL (μg) | Encapsulation efficiency (%) | Dried particle weight (mg) | Loading capacity (%) |
| --- | --- | --- | --- | --- | --- | --- |
| 1 | 1 | 20 | 929.78 | 92.98 | 17.52 | 5.31 |
| 2 | 1 | 20 | 982.49 | 98.25 | 18.75 | 5.24 |
| 3 | 1 | 20 | 998.27 | 99.83 | 16.20 | 6.16 |
| Mean | 1 | 20 | 970.18 | 97.02 | 17.49 | 5.57 |


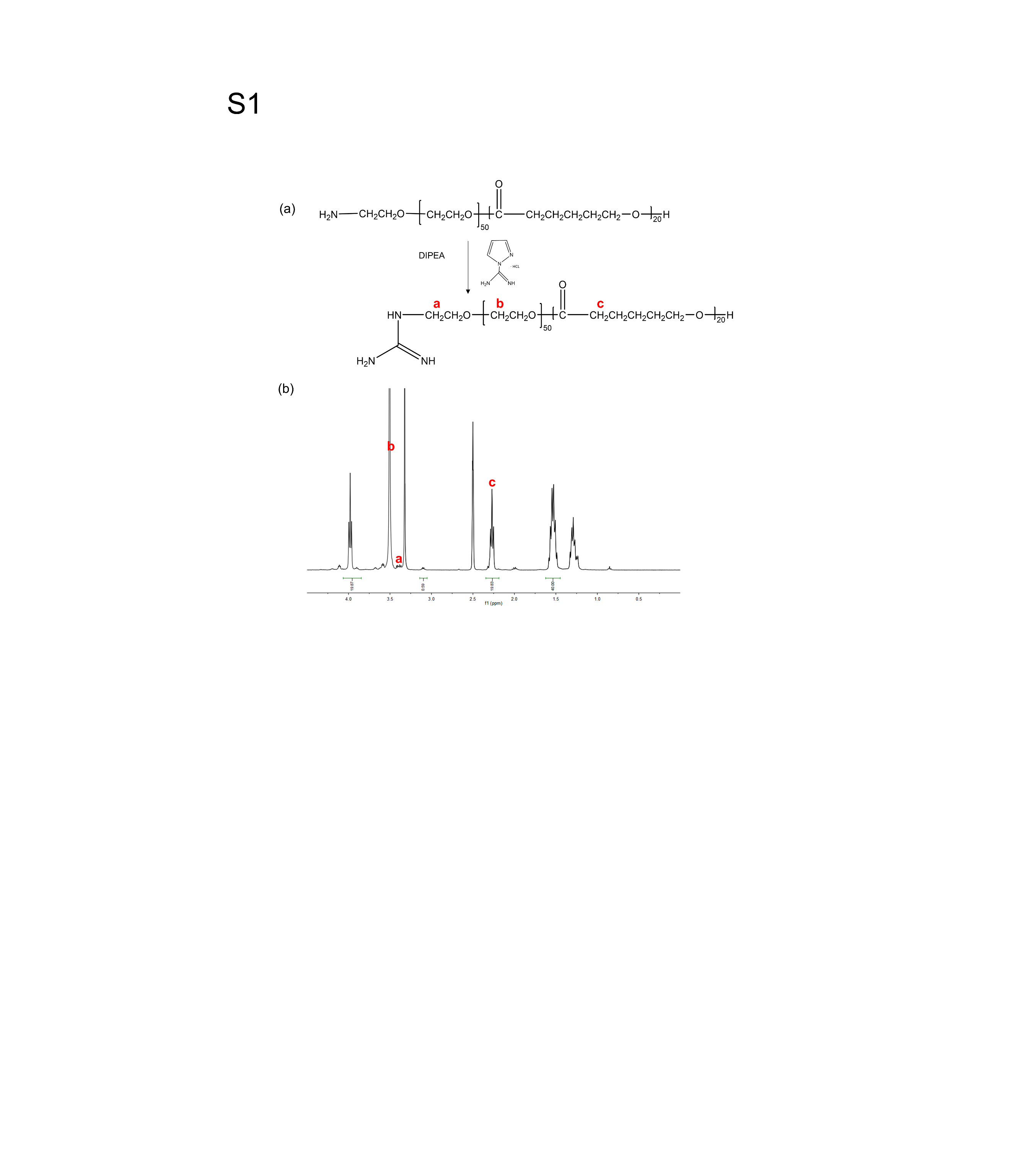


**Fig. S1** ^1^H nuclear magnetic resonance spectra of G-PEG-PCL.


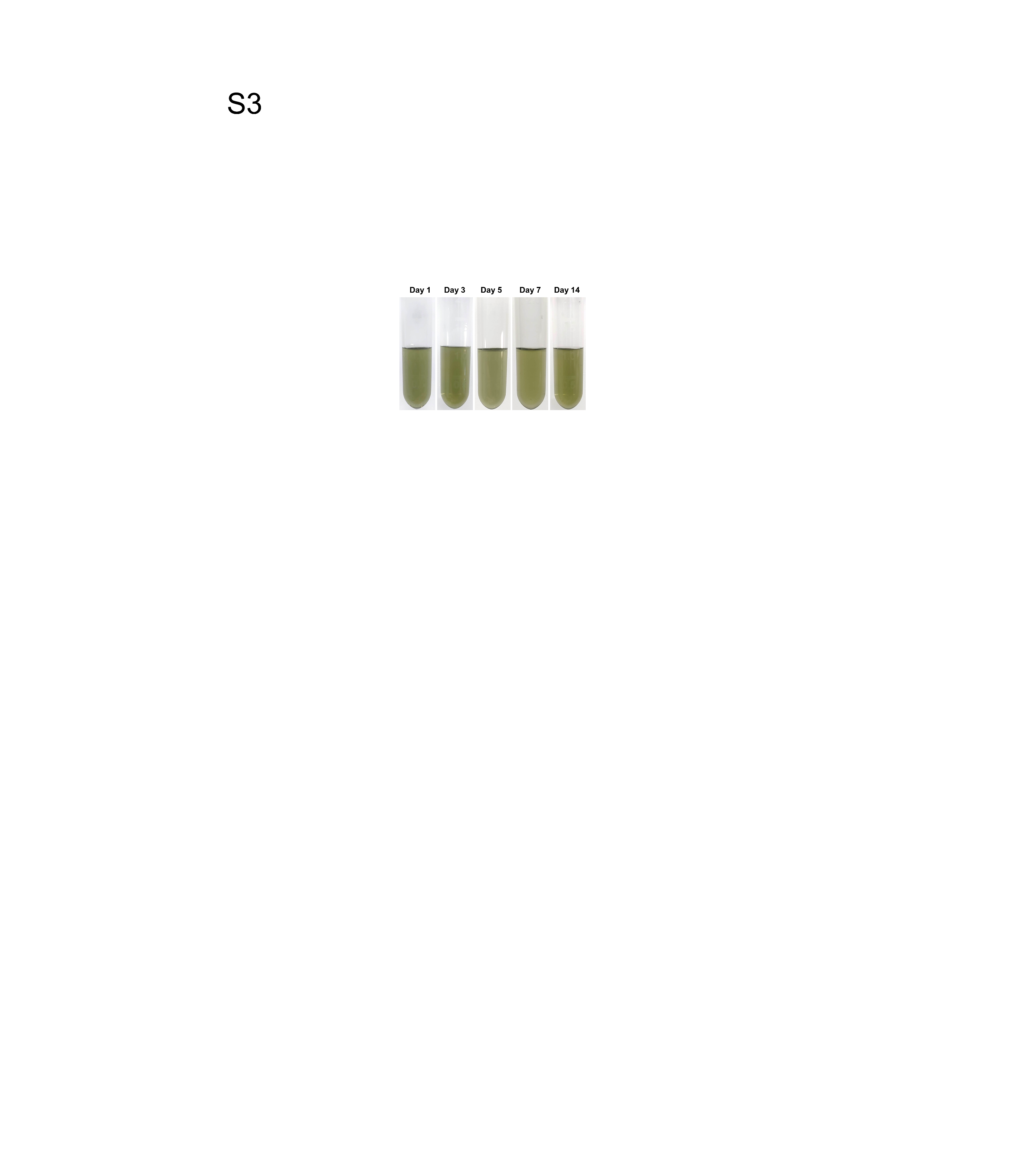


**Fig. S2** The stability of CGP during 14 days.

**Fig. S3** Ultraviolet-visible absorption spectra of free Ce6, CPP, and CGP.

**Fig. S4** Fluorescence emission spectra of free Ce6, CP, and CGP

**Fig. S5** Total ROS generation profiles of different groups over various durations. Data are presented as mean ± SEM, n = 3, **** p ≤ 0.0001. Comparison between CGP+Laser versus other groups.


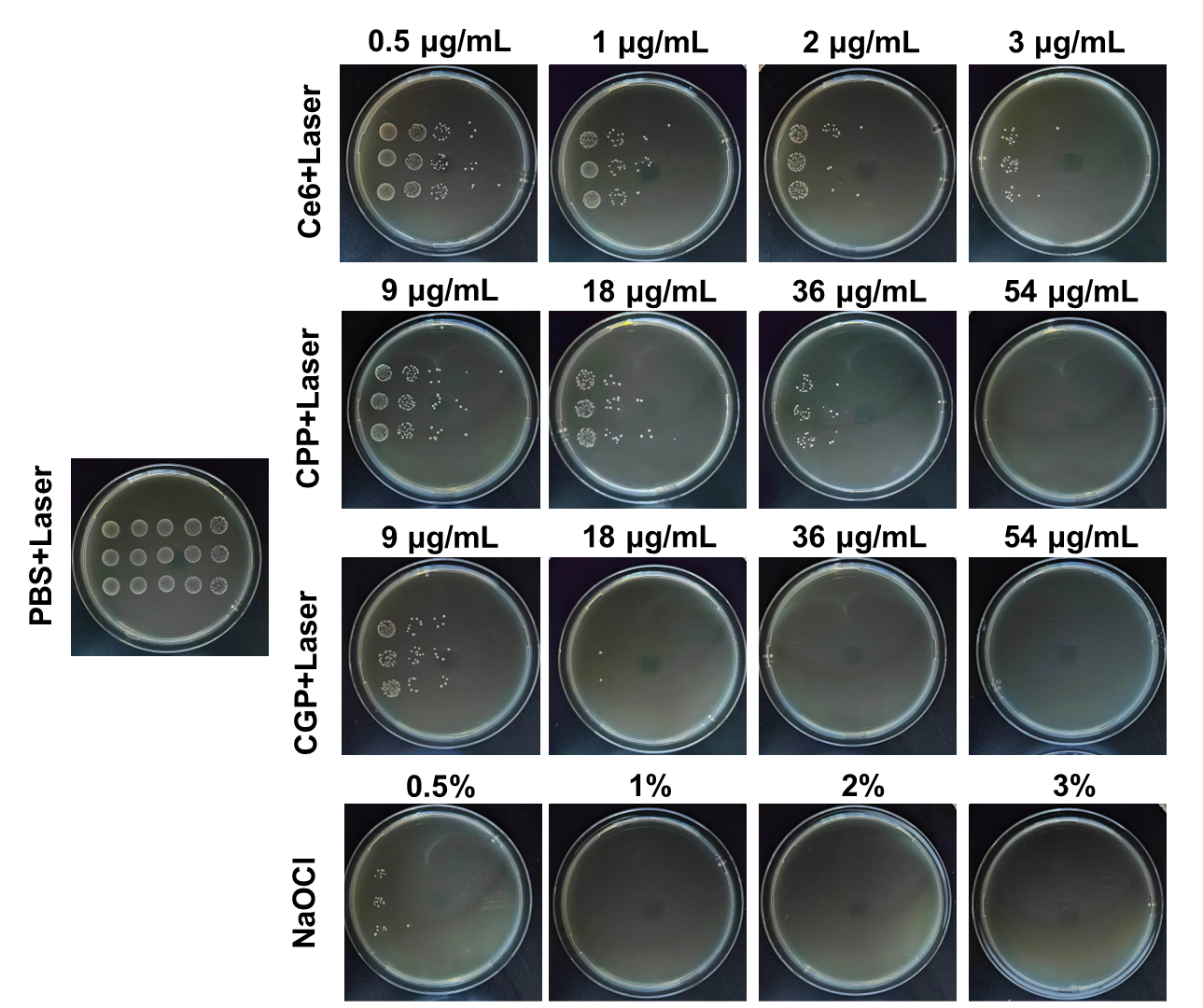


**Fig. S6** Representative images of plate samples of E.f after various treatments.

**Fig. S7** bacterial viability of E.f after various treatments.


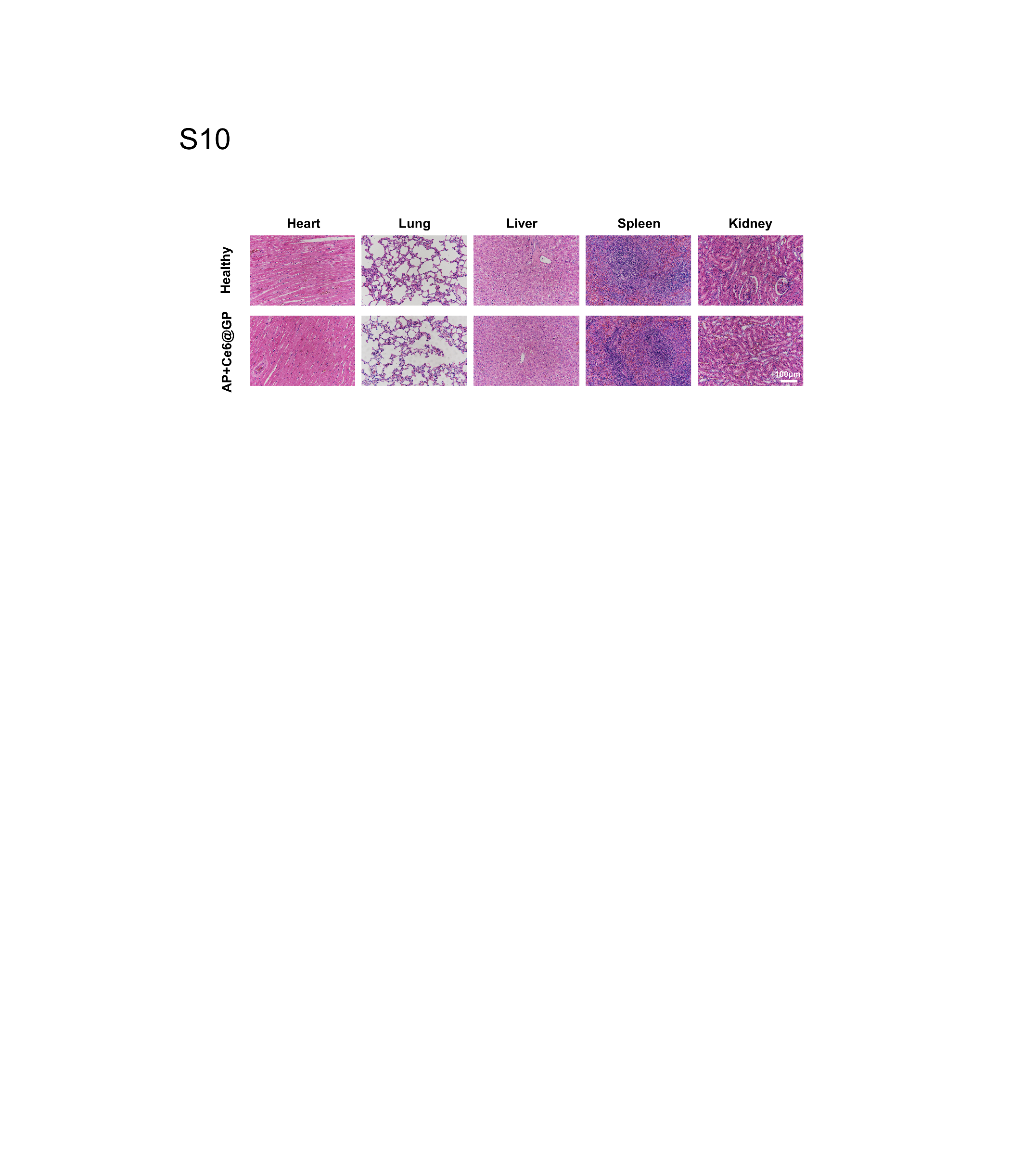


**Fig. S8** HE staining of heart, lung, liver, spleen, and kidney in healthy group and CGP+Laser treated AP group.
